# Much Ado about Prompting: LLM classification of text messages from experiments

**DOI:** 10.1371/journal.pone.0354757

**Published:** 2026-08-04

**Authors:** Can Çelebi, Stefan P. Penczynski

**Affiliations:** 1 Vienna Center for Experimental Economics (VCEE), University of Vienna, Oskar-Morgenstern-Platz 1, Vienna, Austria; 2 School of Economics and Centre for Behavioural and Experimental Social Science (CBESS), University of East Anglia, Norwich Research Park, Norwich, United Kingdom; A’Sharqiyah University, OMAN

## Abstract

Researchers who classify text with large language models often possess codebooks written for human annotators. We ask how these codebooks can serve as prompts. Using three codebooks from experimental economics (one on promise classification, two on strategic thinking), we vary the level of information in the prompt, then vary its formatting, framing and wording at the lowest and highest information levels, across two proprietary and two open-weight models. Used as prompts, the codebooks reach 82–88% agreement with human annotators across the three tasks. On the recognition-heavy task (promise classification), model choice accounts for most of the variation in accuracy; on the learning-heavy tasks (strategic thinking classification), the level of detail in the classification instructions carries comparable weight. These information components partly substitute for one another, whereas model reasoning does not reliably compensate for missing content and yields little or no improvement once the content is present. Larger models make better use of additional information and are more robust to formatting, framing, and wording of the prompt, while smaller models can be hurt by extra information and are more sensitive to how the information is presented. Our results advocate for a shift in focus from prompt engineering techniques (formatting, framing, reasoning, etc.) to the content of the prompt: preparing instructions as one would for human annotators, with detailed context, category definitions, and examples.

## 1. Introduction

In text classification tasks, researchers require a *codebook*, either written from scratch or adapted for a new setting from an existing one (For example, the promise classification codebook developed by [1] for a trust game was later adapted by [2] for a public goods game.) to instruct *human* annotators. These codebooks lay out the underlying context and theory for the classifications, and provide detailed category definitions and annotated examples for each category. When researchers turn to large language models (LLMs) to perform the same task, a natural question arises: can the codebook prepared for human annotators be used directly as an LLM prompt?

The literature offers guidance on how to prompt “effectively”. Researchers are advised to structure their prompts with markdown headers and bullet lists [3–5], to assign role personas [6–8], to provide background context and label descriptions [9,10], to include worked examples [11], and to restructure the codebook for the model [12]. However the majority of this advice draws on early prompting research on GPT-2 and GPT-3 [13,14], where models were less capable at following instructions and prompts were typically short, one- or two-sentence task descriptions rather than detailed codebooks [15]. Following these recommendations requires effort to transform the codebook into something “optimised” for the model, yet whether they still apply to today’s frontier models, or to substantially more detailed prompts, is unclear.

Apart from the informational content of a prompt, there is also the question of how that content is presented: choices about formatting (markdown vs. plain text, bullet lists vs. prose), framing (role persona, task preamble), and phrasing (specific word choices, sentence structure). We refer to these collectively as *surface variations*: changes that alter how the content is presented without changing the informational detail of what it communicates about the classification task. Ideally, classification outcomes should be robust to surface variations. Yet, several studies have shown that LLM outputs can be sensitive to them, producing significant variance in classification outcomes [16,17]. We refer to this sensitivity as *prompt brittleness*: the dependence of classification outcomes on surface variations [18]. If models are brittle in this way, then any particular prompt formulation may not be robust, and iterating on phrasing and format may not lead to a reliably optimal prompting solution.

In this paper we take three classification codebooks from experimental economics and decompose each into its distinct informational components: classification instructions, experiment context, theoretical background, and worked examples. We then independently vary the level of informational detail in each component in a factorial design and compare classification performance within and across two proprietary (GPT 5.4, GPT 5.4-nano) and two open-weight models (Qwen 3.5 397B, Qwen 3.5 9B). At the lowest and highest levels of informational detail, we additionally investigate the effect of surface variations (formatting, framing, wording) as well as the use of model reasoning on performance across the four models.

Our central finding is that a codebook transcribed into a prompt with minimal modification reaches 82–88% agreement with humans across the four models tested. Performance (Throughout the paper, *performance* (or *accuracy*) refers to the agreement rate between the model’s classification and the ground truth, defined as the label on which two independent human annotators agreed. See Section 4 for details.) is driven primarily by the level of informational detail in the classification instructions: experimental context, theory, category definitions, and examples. These components partly compensate for one another when some or all others are missing, and partly complement one another when combined. This validates the use of detailed codebooks prepared for human annotators as prompts for LLMs.

The benefits of using a codebook as a prompt on model performance, however, depend on both the task and the model. Strategic thinking classification (a learning-heavy task) benefits substantially from detailed instructions, whereas promise classification (a recognition-heavy task) benefits more from model size than from prompt content. Larger models, GPT 5.4 and Qwen 3.5 397B, make better use of additional detail (full codebook content), whereas for smaller models it provides no significant benefit, and can even hurt performance.

Surface variations have no significant effect for GPT 5.4 and only marginal effects for GPT 5.4-nano, while the open-weight Qwen models display some degree of prompt brittleness. However, even in cases where surface variations significantly affect performance, their effects have no systematic direction: the same formatting, framing, or wording change can improve performance in one case and reduce it in another. These surface variations are therefore either inconsequential or unreliable as tools for improving performance.

Enabling model reasoning, or increasing its degree gradually from none to extremely high in the GPT models, does not reliably improve performance. Reasoning is at best a partial substitute when the prompt lacks the informational detail of a codebook, and it is not a complement once detailed context, theory, category definitions, and examples are provided. Furthermore, for GPT models, at high reasoning settings (high and extremely high), reasoning either provides no benefit or significantly worsens performance relative to the no reasoning baseline.

These results contribute to the LLM text classification literature in three ways. First, we show that once content unrelated to the classification task is filtered out, an existing codebook prepared for human annotators can be used as-is to serve as a reliable prompt for LLMs. Second, we are the first to jointly examine three dimensions of LLM text classification: informational detail of the prompt, prompt engineering (formatting, framing, phrasing, and reasoning), and model scale. We find that informational detail dictates whether surface variations matter, while model scale dictates how much they matter. Lastly, this joint perspective reconciles conflicting prompting recommendations and explains the documented issue of prompt brittleness in the literature. Our results indicate that prompt brittleness is likely an artifact of classification done with underspecified prompts or relatively small-scale models (e.g., Qwen 9B).

The practical recommendation is to pair a frontier model with a well-defined codebook. By well-defined, we mean a codebook that provides the level of information a researcher would need to give human annotators: the relevant context, underlying theory, category definitions, and examples. Researchers can then spend their effort on getting this informational detail right, rather than on prompt engineering techniques.

## 2. Related work

LLMs are increasingly used for text classification in the social sciences, with recent work confirming that LLMs can achieve inter-rater reliability comparable to trained coders across multiple disciplines [19–22]. As adoption grows, so does the question of how best to instruct the model. Studies testing this question produce apparently conflicting recommendations: formatting matters substantially [3,16]; detailed codebooks help [23–25]; detailed codebooks can hurt for small models [26]; personas are recommended [4,6]; personas are ineffective or harmful [27–30].

A useful organising principle comes from [15], who distinguish *underspecified* prompts — which provide little task description and no label constraints — from *well-specified* prompts that include explicit instructions (Pecher et al. establish this distinction at the lower end of the specification range: their underspecified prompts are essentially raw completion cues of around five words (e.g., “{text}\nSentiment:”), while their well-specified prompts are brief task descriptions of around 25 words (e.g., “Determine the sentiment of the following text. Use one of these labels: negative; positive.”). Our design space complements theirs by extending further into richer specification levels: our minimal prompt is already comparable to their well-specified condition, and our verbatim codebook prompts run to 1,000–1,500 words including theoretical frameworks, category definitions, and worked examples. Building on their finding that any task description beats no task description, we show that the further richness of a full researcher codebook continues to add value.). On underspecified prompts, model predictions are near-chance and any surface variation can shift outcomes; on well-specified prompts, predictions are robust and surface variations become noise.

This distinction reconciles both the conflicting recommendations regarding optimal prompt design [4,23,26,27] and the broader phenomenon of prompt brittleness [16,17,31,32]. Studies reporting significant performance fluctuations due to surface variations use underspecified prompts, models at smaller scales, or both. Although this framework may not resolve all conflicting findings in the literature, it aligns closely with the empirical results most comparable to our own (See S12 Appendix for further discussion.).

## 3. Research questions

In our setting, a codebook contains four functional components: classification instructions defining each category, experiment context describing the game mechanics, theory context laying out the formal theoretical framework, and worked examples. This structure is characteristic of experimental economics, where classifications are often grounded in a formal model. Codebooks in other domains may omit the theory layer, but classification instructions and examples are likely present in any well-specified codebook. How much of this detail a model actually needs depends on what it already knows from pre-training. Promise classification (*P*) is likely a *recognition-heavy* task: the concept is intuitive, and related notions of commitment and promise-keeping appear widely in natural language [33,34]. Strategic thinking classification (*L*) is a *learning-heavy* task: level-*k* reasoning is a technical construct specific to behavioural game theory [35], unlikely to be prevalent in pre-training corpora. We therefore ask:

**RQ1.** *For a given task, can existing codebooks serve as effective LLM prompts, and which of their components drive classification performance?*

Even if the codebook content provides sufficient detail, how it is presented (formatting, framing, phrasing) and whether the model reasons through the task may still affect classification. The literature offers recommendations on these choices, typically without controlling for the level of informational detail present in the prompt. We test each intervention at both high and low levels of informational detail. This allows us to ask whether these “prompt engineering” techniques improve classification in their own right, or whether they only appear to help when the prompt lacks sufficient detail and the techniques partially fill that gap. This leads to our second question:

**RQ2.** *Do prompt engineering choices — formatting, framing, wording, and reasoning — improve classification beyond what the codebook content provides?*

The answers to both questions may depend on the model. A large model may extract value from a verbose codebook that overwhelms a smaller one; a smaller model may depend on prompt engineering choices that make no difference for a frontier model. We approximate model scale by total parameter count where it is disclosed; for the proprietary GPT models, where counts are undisclosed, we treat the ‘nano’ suffix as marking GPT 5.4-nano as smaller than GPT 5.4. We evaluate four models at two scale tiers within each family: GPT 5.4 and GPT 5.4-nano (proprietary), Qwen 3.5 397B and Qwen 3.5 9B (open-weight). This motivates our third question:

**RQ3.** *How does model scale affect classification performance and moderate the sensitivity to prompt content and design?*

## 4. Data

We use classification codebooks from three experimental economics studies, investigating two distinct classification tasks: identifying promises and inferring strategic reasoning levels. The *promise* task (*P*) classifies open-ended chat messages from a public goods game [2] as promise or empty talk; we use the 1,494 player-level utterances (drawn from 719 chat messages) on which both annotators agreed. The *level-k* tasks classify statements by strategic reasoning level: *L*_I_ classifies 493 messages from a voting game [36] into levels 0–3, and *L*_II_ classifies 851 messages from a set of coordination games [37] into levels 0–5.

In all three tasks, two human annotators independently classified each item; ground truth is defined by the instances on which both annotators agreed. An item is scored as correct if the model’s prediction matches this agreed label, and a model’s accuracy is the proportion of items it classifies correctly (For *L*_I_ and *L*_II_, annotators were incentivised via a coordination game — paid based on agreement with each other — and informed of their disagreements between two rounds of classification. [38] show that this incentive structure makes annotators more responsive to fine-grained changes in classification instructions. The *P* annotations were produced without such incentivisation).

## 5. Design

**Fig 1:**
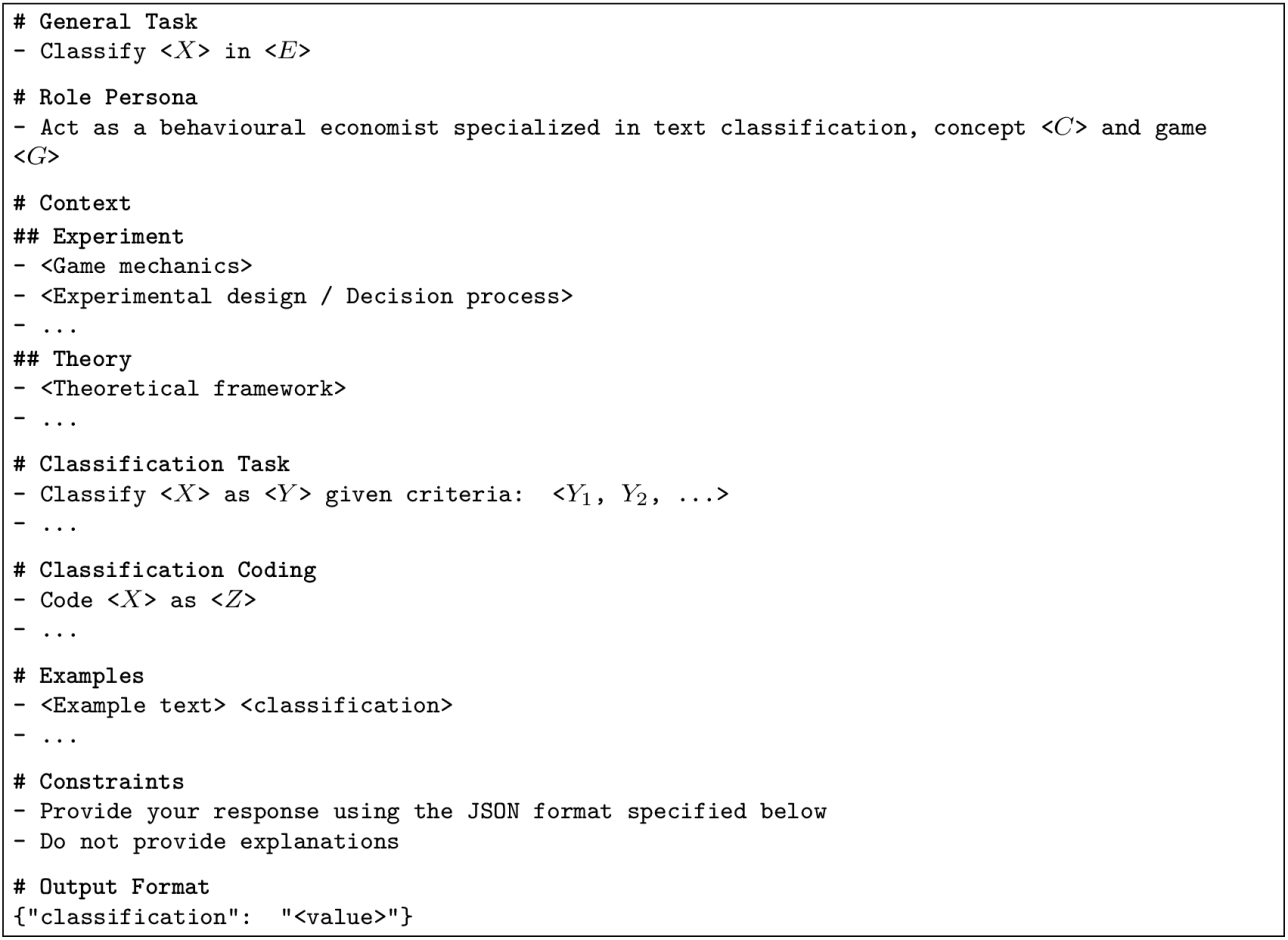
Prompt template. Theory subsection is included only for *L*-tasks. Examples are included only in *n*-shot conditions. All prompts use markdown formatting with itemized lists.

All classification tasks use a standardized prompt template (Fig 1), informed by existing prompting guidelines and empirical findings on effective prompt design (Each template component is motivated by specific findings in the prompting literature. See S11 Appendix for the full rationale and citations.). The template begins with a general task description and a role persona, followed by experiment context, theory context, and classification instructions drawn from the codebook. Worked examples from the codebook are included in the *n*-shot conditions. The experiments that follow test each dimension of the template and their interactions, with the exception of the output format sections (constraints, classification coding, and output format), which are held constant across all conditions (Constraints and output format enforce structured JSON responses, which has been shown to improve classification accuracy [39] and lets the classification label be read directly from the response without any string parsing.).

In total, across all experiments, approximately 703,000 classification requests were submitted to the API (703,253 calls; 702,809 successful; 0.06% error rate), covering 1,182 unique model–task–condition combinations across 4 models. Experiments were conducted between 24 March and 6 April 2026.

### 5.1. Codebook-to-prompt pipeline

We convert each human classification codebook into a set of LLM prompts through a four-step pipeline applied uniformly to all four tasks. All LLM-assisted steps use Claude Opus 4.6.

**Step 1: Filter.** The raw codebook is processed with a structured filtering prompt (see S10 Appendix) that removes content relevant only to human annotators in a laboratory setting: greetings, payment details, software instructions, data-entry guidance, meta-instructions, and deadline warnings. Classification-relevant content — experiment mechanics, category definitions, and worked examples — is retained verbatim. As a light reframing, second-person laboratory instructions (“Your task is to…”) are converted to third-person descriptions (“Each participant…”). No information is added or paraphrased beyond this reframing.

**Step 2: Separate.** The filtered codebook is manually divided into three named sections: *experiment context* (game mechanics, payoffs, communication protocol), *theory context* (the level-*k* framework, L-tasks only), and *classification instructions* (category definitions and assignment rules). Worked examples are extracted into a separate file for use in few-shot conditions. This separation allows each section to be independently varied in subsequent experiments.

**Step 3: Compress.** Each section is iteratively compressed to two intermediate levels (C1 at approximately 50–60% and C2 at approximately 25–30% of the verbatim word count) using an LLM with a detailed compression prompt (see S10 Appendix). The prompt is applied twice in sequence (verbatim → C1, C1 → C2) and instructs the model to only remove content — never to add, infer, or paraphrase — while preserving a task-specific list of key technical terms at every level. Table 2 shows that embedding cosine similarity to the verbatim text remains above 0.80 at all compression levels, and that all key terms are preserved across all tasks and all compression steps.

**Step 4: Assemble.** At runtime, prompt variants are assembled from their component sections. The assembler concatenates sections in a fixed order (framing header, experiment context, theory context, classification instructions, examples) and applies the requested content level and format treatment, producing a single system prompt. The text to classify is provided in the user prompt (See S8 Appendix for details.).

### 5.2. Models

We evaluate four models: two proprietary and two open-weight (Table 1). The GPT 5.4 pair (5.4 and 5.4-nano) represents a frontier closed-source family at two capability tiers; parameter counts are not disclosed. The Qwen 3.5 pair (397B and 9B) represents a state-of-the-art open-weight family at two known scales — 17B active parameters (MoE) and 9B (dense) — enabling a within-family scale comparison. GPT models are accessed through Azure OpenAI Service; Qwen models through Together AI and OpenRouter.

**Table 1:** Models evaluated in this study.

Model	Company	Total	Active	Architecture	Released	Provider
GPT 5.4	OpenAI	—	—	closed	Mar 2026	Azure OpenAI
GPT 5.4-nano	OpenAI	—	—	closed	Mar 2026	Azure OpenAI
Qwen 3.5 397B	Alibaba	397B	17B	MoE	Feb 2026	Together AI / OpenRouter
Qwen 3.5 9B	Alibaba	9B	9B	dense	Feb 2026	Together AI / OpenRouter

*Note:* Active params = parameters active per forward pass (equal to total for dense models). GPT parameter counts are not disclosed. GPT models support reasoning at multiple effort levels; Qwen models support a binary reasoning toggle.

### 5.3. Experiments

We conduct six experiments, each targeting a different dimension of prompt design. All experiments use the same items and models, enabling paired comparisons throughout. We represent each prompt configuration as a tuple (*c*, *x*, *t*, *e*), where *c* is the classification instruction level, *x* is the experiment context level, *t* is the theory context level (*L*-tasks only), and *e* is the example condition. Subscripts denote detail: verbatim (*V*), compressed (*C*_1_, *C*_2_), or absent (*N*) for context and theory; verbatim (*V*) or simple (*S*) for classification instructions; zero-shot (∅) or *n*-shot (*n*) for examples. Two anchoring configurations are used throughout:

- **Verbatim** $\left(c_{V},\;x_{V},\;t_{V},\;\emptyset \right)$: the full codebook content — classification instructions, context, and theory — transcribed verbatim, without examples.
- **Minimal** $\left(c_{S},\;x_{N},\;t_{N},\;\emptyset \right)$: one-line category descriptions only, with no context, theory, or examples.

#### 5.3.1. Content ablation.

Each codebook is decomposed into three independently varied information sources: classification instructions (verbatim or simple), experiment context (V/C1/C2/N), and theory context (*L*-tasks only; V/C1/C2/N). Crossing these factors yields 8 variants per *P*-task (4×2, no theory) and 32 per *L*-task (4×4×2).

Classification instructions are tested at two levels. *Verbatim* (*V*) transcribes the codebook’s category definitions in full — typically 6–15 bullet points per category covering inclusion criteria, edge cases, and boundary rules. *Simple* (*S*) reduces codebook’s category definitions to a single definitional sentence (e.g., “Classify the player’s level of strategic thinking as 0, 1, 2, or 3”), retaining only 3–11% of the verbatim word count.

Experiment context and theory context are each compressed to two intermediate levels (C1, C2) using the procedure described in Section 5.1. Table 2 shows that embedding cosine similarity to the verbatim text remains above 0.80 at all compression levels and all key terms are retained (Full cosine similarities for all tasks are in S1 Appendix; the full factorial design is in S6 Appendix.).

**Table 2:** Semantic retention by prompt level (*L*_I_).

Source	Level	Cosine	Words	Key terms
Exp. context	V	1.000	293 (100%)	10
	C1	0.857	174 (59%)	10/10
	C2	0.831	97 (33%)	10/10
	N	—	0 (0%)	—
Theory	V	1.000	735 (100%)	9
	C1	0.901	374 (51%)	9/9
	C2	0.804	158 (21%)	9/9
	N	—	0 (0%)	—
Cls. instructions	V	1.000	—	—
	S	0.724	— (3%)	—

*Note:* Cosine similarity computed against the verbatim original using the text-embedding-3-large model of OpenAI. Key terms = task-critical tokens (game labels, payoff numbers, category names) enforced by the compression prompt. Word counts shown for *L*_I_; other tasks in S1 Appendix.

#### 5.3.2. Formatting and framing.

**Format.** Five treatments are applied to identical textual content: markdown with headers and bullet lists (baseline), titles with bullet lists, titles with prose paragraphs, flat bullet lists without headers, and plain unformatted text. Each format is tested at both verbatim and minimal content levels, yielding 5 formats × 3 tasks × 2 content levels = 30 conditions per model (see S6 Appendix for details).

**Framing.** Two framing elements are tested: a general task description (GT) that names the experimental domain (e.g., “classify messages from a coordination game”) and a role persona (RP) that assigns a domain identity (e.g., “behavioral economist with expertise in level-*k* reasoning”). Three removal conditions (GT only, RP only, Neither) are compared against the full-framing baseline at both content levels, yielding 4 × 3 × 2 = 24 conditions per model (see S6 Appendix for details).

#### 5.3.3. Examples.

Each codebook’s worked examples are added to the prompt in an *n*-shot format. The number of examples is determined by what each codebook provides: 21 for *P* (10.5 per label), 13 for *L*_I_ (3.25 per level), and 5 for *L*_II_ (1.25 per level). Examples are tested at both content levels (minimal and verbatim), enabling a direct comparison of content versus examples as alternative sources of classification information, yielding 2 example conditions × 2 content levels × 3 tasks = 12 conditions per model.

#### 5.3.4. Reasoning.

Model reasoning is enabled at multiple effort levels. GPT models support five levels (off, low, medium, high, extra-high); Qwen models support a binary toggle (on/off). Reasoning is crossed with content level (minimal/verbatim) and example provision (0-shot/*n*-shot), yielding 5×2×2×3=60 reasoning variants per GPT model and 2×2×2×3=24 per Qwen model (baselines shared with other experiments) (see S6 Appendix for details).

### 5.4. Metrics

LLMs are not fully deterministic: even with the same prompt, the same input text, and temperature set to 0, repeated runs can produce different classifications. This arises from numerical non-associativity in floating-point operations during inference, which makes token selection sensitive to hardware-level variation when the gap between the top candidates is small [40]. In practice, studies running the same prompt on the same items 30–50 times report that 8–12% of classifications change across runs, with more complex tasks showing even higher variability [41,42]. A few-point accuracy gap between two prompts can therefore reflect run-to-run noise rather than a genuine treatment effect. We address this by using McNemar’s test throughout: each comparison pairs the *same items* under two conditions, and the test asks whether the items on which the two conditions disagree split asymmetrically, with one condition systematically more often correct than the other. This paired design controls for item difficulty and isolates the treatment effect from run-to-run variation. Significance is assessed at *p* < 0.001 throughout. Accuracy is computed per item as described in Section 4; all accuracy estimates are reported with 95% Wilson confidence intervals [43]. Additionally, instead of accuracy, S13 Appendix reports tables and decompositions in terms of the support-weighted macro F1 score with paired bootstrap confidence intervals; the qualitative findings are unchanged, and the F1 replication is discussed there alongside the accuracy comparison.

## 6. Results

### 6.1. Content

Table 3 reports accuracy under the minimal and the verbatim prompt. On *P*, the verbatim prompt yields no significant improvement over minimal: GPT 5.4 moves from 73.2% to 74.2%, and the other models show similarly small or even negative changes. Promise classification thus appears to be recognition-heavy: the model can often map the label to the relevant concept without needing further information (A variance decomposition (see S1 Appendix confirms this: on *P*, model choice accounts for nearly all explained variance (98%), while on the *L*-tasks classification instructions dominate (58% on *L*_I_)). The *L*-tasks, by contrast, are fundamentally learning-heavy. Under the minimal prompt, performance is poor but improves as the information needed to interpret the categories is provided.

**Table 3:** Accuracy (%) at the minimal and verbatim prompts.

	Minimal			Verbatim		
	*P*	*L*_I_	*L*_II_	*P*	*L*_I_	*L*_II_
GPT 5.4	**73.2** [70.9, 75.3]	47.4 [43.0, 51.8]	49.6 [46.2, 52.9]	**74.2** [71.9, 76.3]	81.3 [77.7, 84.5]	**81.0** [78.2, 83.5]
GPT 5.4-nano	60.9 [58.4, 63.4]	**52.5** [48.1, 56.9]	**53.1** [49.8, 56.5]	64.3 [61.8, 66.6]	70.2 [66.0, 74.1]	68.9 [65.7, 71.9]
Qwen 397B	**70.5** [68.1, 72.7]	50.1 [45.7, 54.5]	43.6 [40.3, 46.9]	66.2 [63.8, 68.6]	**83.0** [79.4, 86.0]	74.6 [71.6, 77.4]
Qwen 9B	64.1 [61.7, 66.5]	**53.1** [48.7, 57.5]	38.8 [35.6, 42.1]	63.3 [60.8, 65.7]	70.8 [66.6, 74.6]	64.4 [61.1, 67.5]

*Note:* 95% Wilson CIs below each estimate. **Bold** = highest value per task within each content level; tied values (McNemar p≥0.001) share bold.

**Fig 2:**
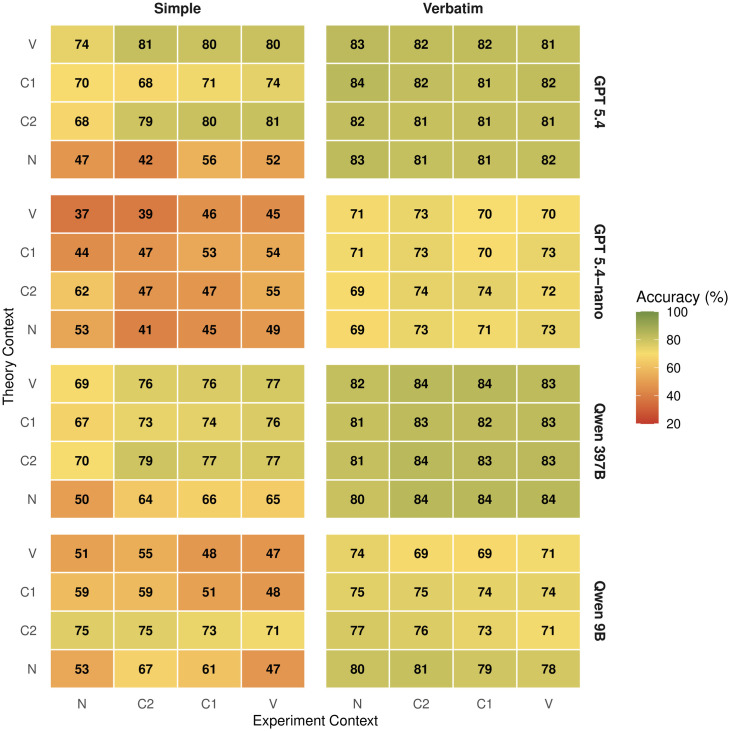
*L*_I_ information surface. Accuracy (%) by experiment context (x-axis) and theory context (y-axis), separately under simple (left) and verbatim (right) classification instructions. Context levels: N = none, C2 ≈ 25%, C1 ≈ 50%, V = verbatim.

Fig 2 shows how theory and experiment context interact on *L*_I_ across different levels of classification detail (*L*_II_ surfaces in S1 Appendix). Under simple classification instructions in the left column, theory contributes substantially on its own, whereas experiment context alone either hurts performance or marginally benefits it. Once theory is present, however, experiment context becomes effective, indicating that the two components complement each other: theory helps define the categories, while experiment context helps map them onto the task. Together, they can partially substitute for missing classification instructions. By contrast, under verbatim classification instructions in the right column, the surface is largely flat for GPT 5.4 and Qwen 397B, with only small positive differences across context conditions. This suggests that theory and experiment context matter primarily when classification instructions are under-specified.

Model scale further moderates this pattern. GPT 5.4 and Qwen 397B benefit from additional content when classification instructions are absent and still benefit marginally when classification instructions are present. Smaller models, by contrast, perform best when classification instructions are present but context is limited or absent, reaching their highest accuracy at the lowest (C2) compression level (Fig 2). This indicates that they are less able to make effective use of the full contextual information (see S1 Appendix for *L*_II_ information surface).

**Result 1.** *Experiment and theory contexts partially complement each other and partially substitute for classification instructions. Larger models benefit from richer content, whereas smaller models perform better with less.*

### 6.2. Formatting and framing

*Formatting.* On verbatim prompts, switching from markdown to any of the four alternative formats produces almost no significant effects for any model (Table 4, left half). On minimal prompts, Qwen 9B shows scattered significance (.33 positive, .17 negative) but with no consistent direction; the other three models remain largely unaffected.

**Table 4:** Formatting and framing: proportion of significant effects.

	Formatting				Framing			
	Minimal		Verbatim		Minimal		Verbatim	
	+	–	+	–	+	–	+	–
GPT 5.4	.17	.08	.00	.08	.33	.11	.33	.00
GPT 5.4-nano	.08	.00	.08	.00	.33	.22	.00	.00
Qwen 397B	.08	.08	.00	.00	.11	.44	.00	.00
Qwen 9B	.33	.17	.00	.00	.44	.00	.00	.11

*Note:* Proportion of all comparisons that are significantly positive (+) or negative (-) at *p* < 0.001. Formatting: 4 treatments vs. markdown baseline (12 per cell: 4 formats × 3 tasks). Framing: 3 conditions (both, GT only, RP only) vs. no-framing baseline (9 per cell: 3 conditions × 3 tasks). Mean effects and dispersion in S2 Appendix.

*Framing.* On verbatim prompts, framing effects are again negligible (Table 4, right half). On minimal prompts, the pattern is model-dependent: Qwen 9B shows .44 significant positive effects (driven by domain cues in the general task description that are absent from the sparse prompt), while Qwen 397B shows .44 significant negative. For GPT 5.4, the few significant framing effects are driven entirely by the general task description (GT) rather than the role persona (On verbatim prompts, GPT 5.4 shows three significant positive effects, all GT-driven: GT only on *P* (+2.9 pp), GT only on *L*_II_ (+3.6 pp), and Both on *P* (+2.8 pp). The GT sentence names the domain (“public goods game”, “coordination game”), providing a keyword anchor even when the full codebook is present. Role persona alone never reaches significance on any model on verbatim prompts. See S2 Appendix for details).

**Result 2.** *Neither formatting nor framing reliably affects classification accuracy. On verbatim prompts, effects are negligible. On minimal prompts, significance increases but the direction is inconsistent across models and tasks.*

### 6.3. Examples

Table 5 decomposes the contributions of content and examples (We also varied the in-prompt format of the examples (markdown headers, XML tags, plain delimiters, arrow notation, multi-turn conversation); design and full results in S3 Appendix.) for GPT 5.4. Examples alone produce large accuracy gains on every task ($\Delta_{\text{Ex}}=+16.6$ pp on *P*, + 37.4 pp on *L*_I_, + 20.4 pp on *L*_II_), comparable to or larger than the gain from adding verbatim content alone. Content and examples convey overlapping information about what the categories mean, making them partial substitutes. Once verbatim content is already in the prompt, the value of additional examples scales with the number of examples per label the codebook supplies (*P* provides 21 worked examples (10.5 per label), *L*_I_ 13 (3.25 per level), and *L*_II_ 5 (1.25 per level); see S3 Appendix. A task-intrinsic effect — *P* being more pattern-recognition and *L* more theory-laden — may also contribute to this monotonic decline; our design does not let us isolate it from the example-count effect.): $\Delta_{\text{Ex}|\text{Content}}$ is + 14.0 pp on *P*, + 2.6 pp on *L*_I_, and a non-significant +0.7 pp on *L*_II_. The same monotonic pattern appears under weighted F1 and across all four models (See S13 Appendix for details.).

**Table 5:** GPT 5.4: effect of examples.

	*P*	*L*_I_	*L*_II_
$\Delta_{\text{Ex}}$	+16.6^*^	+37.4^*^	+20.4^*^
$\Delta_{\text{Ex}}-\Delta_{\text{Content}}$	+15.6^*^	+3.4	−10.9^*^
$\Delta_{\text{Ex}|\text{Content}}$	+14.0^*^	+2.6	+0.7
$\Delta_{\text{Content}|\text{Ex}}$	−1.6	−0.8	+11.6^*^

*Note:* ^*^ = McNemar *p* < 0.001. All values in pp. $\Delta_{\text{Ex}}$: gain from adding examples to the minimal prompt. $\Delta_{\text{Ex}|\text{Content}}$: gain from adding examples to the verbatim prompt. $\Delta_{\text{Ex}}-\Delta_{\text{Content}}$: minimal+examples minus verbatim+zero-shot (positive = examples alone outperform content alone). $\Delta_{\text{Content}|\text{Ex}}$: verbatim+examples minus minimal+examples (positive = content adds value on top of examples).

**Result 3.** *Examples alone produce large accuracy gains across all tasks. Content and examples are partial substitutes. The accuracy gain from examples given content scales with the example count.*

Table 6 represents what a researcher obtains by faithfully transcribing the codebook with its worked examples (with the minor LLM-assisted filtering described in Section 5.1). On *P* and *L*_I_, the open-weight Qwen 397B matches the proprietary GPT 5.4 (McNemar p≥0.001), and even Qwen 9B reaches 83% on *L*_I_. The tasks separate clearly by difficulty: all models achieve 72–88% on *P* and 78–84% on *L*_I_, but *L*_II_ drops to 68–82%, with the gap between frontier and small models widening from ≈2 pp on *L*_I_ to ≈13 pp on *L*_II_.

**Table 6:** Accuracy (%) with the full codebook: verbatim content and worked examples.

	*P*	*L*_I_	*L*_II_
GPT 5.4	**88.2** [86.4, 89.7]	**84.0** [80.5, 86.9]	**81.7** [78.9, 84.1]
GPT 5.4-nano	72.0 [69.6, 74.2]	78.3 [74.4, 81.7]	70.7 [67.6, 73.7]
Qwen 397B	**86.7** [84.9, 88.4]	**83.6** [80.0, 86.6]	77.8 [74.9, 80.5]
Qwen 9B	78.5 [76.4, 80.5]	**82.8** [79.2, 85.8]	68.2 [64.9, 71.2]

*Note:* Verbatim content + *n*-shot examples, no reasoning. 95% Wilson CIs below each estimate. **Bold** = highest value per task; tied values (McNemar p≥0.001) share bold.

### 6.4. Reasoning

Can reasoning compensate for missing codebook content — that is, can a model that “thinks harder” overcome an underspecified prompt? Table 7 decomposes reasoning and content effects in the same way Table 5 decomposed examples and content. The answer is no.

**Table 7:** GPT 5.4: content vs. reasoning substitutability.

	*P*	*L*_I_	*L*_II_
$\Delta_{\text{Rea}}$	+9.5^*^	−11.5^*^	+19.7^*^
$\Delta_{\text{Rea}}-\Delta_{\text{Content}}$	+8.5^*^	−45.4^*^	−11.6^*^
$\Delta_{\text{Content}|\text{Rea}}$	−9.7^*^	+46.7^*^	+11.6^*^
$\Delta_{\text{Rea}|\text{Content}}$	−1.2	+1.2	+0.0

*Note:* ^*^ = McNemar *p* < 0.001. All values in pp, 0-shot only, no examples. For GPT models we test low, medium, high, and extra-high reasoning effort; we report high to match Qwen’s reasoning token output (≈750 tokens). No incremental step from low to extra-high produces a significant improvement (see S4 Appendix). $\Delta_{\text{Rea}}$: minimal+reasoning minus minimal 0-shot. $\Delta_{\text{Rea}}-\Delta_{\text{Content}}$: minimal+reasoning minus verbatim 0-shot (negative = content alone outperforms reasoning alone). $\Delta_{\text{Content}|\text{Rea}}$: verbatim+reasoning minus minimal+reasoning. $\Delta_{\text{Rea}|\text{Content}}$: verbatim+reasoning minus verbatim 0-shot. Remaining models in S4 Appendix.

On *L*-tasks, content alone clearly outperforms reasoning alone ($\Delta_{\text{Rea}}-\Delta_{\text{Content}}$: −45.4 pp on *L*_I_, −11.6 pp on *L*_II_). On *L*_I_, reasoning on the minimal prompt actually *hurts*: $\Delta_{\text{Rea}}=-11.5$ pp, meaning that the model’s attempt to reason without adequate classification instructions produces worse results than no reasoning at all.

The conditional effects are asymmetric: content remains highly valuable when reasoning is active ($\Delta_{\text{Content}|\text{Rea}}$: + 46.7 pp on *L*_I_, + 11.6 pp on *L*_II_), but reasoning adds nothing when content is already present ($\Delta_{\text{Rea}|\text{Content}}$: + 1.2 pp n.s. on *L*_I_, exactly 0.0 pp on *L*_II_). The same pattern holds across all four models (see S4 Appendix): $\Delta_{\text{Rea}|\text{Content}}$ is near-zero on *L*-tasks for GPT 5.4, GPT 5.4-nano, and Qwen 397B, with only Qwen 9B showing a significant effect on *L*_I_ (+10.5 pp). On *P*, smaller models show significant reasoning-given-content gains (GPT 5.4-nano: + 6.5 pp, Qwen 397B: + 8.4 pp, Qwen 9B: + 9.4 pp), suggesting that reasoning can partially compensate for weaker pre-training representations of familiar concepts, but not for missing domain-specific theory.

With reasoning enabled on verbatim prompts at both 0-shot and *n*-shot, GPT 5.4 shows no significant effect on any task at any condition (see S4 Appendix for additional results). Smaller models benefit on *P* at both shot conditions, but on *L*-tasks their gains appear only at 0-shot and disappear once examples are present: examples render the reasoning gains redundant (Adding examples costs ≈ 0.3–0.4× the prompt (input-rate tokens), comparable to low reasoning, yet produces accuracy gains an order of magnitude larger on *L*-tasks. See S9 Appendix for the full cost comparison.).

**Result 4.** *Reasoning cannot substitute for codebook content and does not improve frontier-model classification. On well-specified prompts, smaller models gain from reasoning only on the P-task; their L-task gains at zero-shot disappear once examples are present.*

## 7. Discussion

### 7.1. Summary of findings

Faithful transcription of a codebook — including category definitions, context, theory, and the worked examples it supplies — produces 82–88% agreement with the human-agreed ground truth across our tasks. What drives this accuracy is the level of informational detail in the prompt. Detailed classification instructions are the dominant component, while experiment and theory contexts partially complement each other and together can partially substitute for the classification instructions. Examples on their own also produce large gains. The information they carry partially overlaps with that of the codebook content, making the two partial substitutes: once a verbatim codebook is in place, the value of additional examples scales with how many examples per label the codebook supplies.

How effectively a model utilizes this informational detail depends on both the task and its own scale. On learning-heavy tasks, detailed instructions are essential; on recognition-heavy tasks, model choice explains more of the variation in performance than prompt content. Larger models tolerate surface variation better and extract more value from detailed content. Smaller models are more sensitive, and in some cases, additional detail can even degrade their performance.

How the information is presented is less of a concern for capable models. Smaller models exhibit prompt brittleness, however the direction of their sensitivity to surface variations is inconsistent across tasks and treatments. Similarly, introducing model reasoning does at best partially compensate for missing content, and adds little to no benefit for larger models once detailed content is present.

These results yield an actionable recommendation for applied researchers: shift the focus from prompt engineering to codebook quality. The most reliable classification strategy is to pair a frontier model with a well-defined codebook that provides the informational detail a human annotator would need.

### 7.2. Beyond the codebook

A researcher adapting a human codebook for an LLM might feel that the original is too sparse in places and want to elaborate, adding the kind of explanation, rationale, or worked-out criteria that a careful human reader would also benefit from. We systematically expanded the codebook in this way(We tested three perturbations of the prompt text itself: (i) verbose elaboration (+50–275% words of relevant detail) plus a matched noise control of irrelevant content at the same length; (ii) round-trip back-translation (English → Turkish/German → English); (iii) the raw unprocessed codebook used directly as the prompt. Designs in S6 Appendix; significance tables in S5 Appendix (verbose/noise) and S5 Appendix (paraphrase).) and saw no significant change for GPT 5.4; injecting irrelevant content of matched length had no effect either, suggesting that frontier models filter out what is not useful. Smaller models respond to both treatments at 0-shot, but the effects largely vanish once examples are present. Similarly, paraphrasing produces effects below 1 pp for all models at 0-shot; at *n*-shot, smaller models show occasional sensitivity, again without consistent direction. And the raw unprocessed codebook, including payment instructions and software notes intended for human annotators, performs comparably to the cleaned version for GPT 5.4.

As Sections 1 and 2 discuss, the apparently conflicting reports of prompt brittleness in the literature largely correspond to underspecified prompts or weaker models. Our results extend the point beyond formatting: verbose elaboration, irrelevant noise, paraphrasing, and uncleaned procedural content all fail to move the needle once the codebook is detailed and the model is capable.

A parallel observation comes from compound AI systems: [44] find that prompt optimisation is already a coin flip on tasks where the model’s default behaviour is well-aligned, and argue that instruction tuning and reinforcement learning from human feedback increasingly compress diverse input phrasings into consistent outputs, shrinking the headroom that surface-level optimisation can exploit. We see the same shrinking headroom in the classification setting: much of the prompt-engineering literature was written for earlier, less capable models, and the domain in which it still moves the needle has been narrowing as models improve.

This robustness reflects the quality of our codebooks: experimental-economics codebooks define categories through formal models, give inclusion and exclusion criteria, and provide worked examples grounded in the same theory. Codebooks elsewhere in the social sciences are often sparser, with many labels, short definitions, overlapping categories, and no formal theoretical grounding. In those settings the codebook may genuinely be underspecified, and elaboration or restructuring may be warranted. Our content-ablation results (Section 6.1) make the asymmetry concrete: removing detail from an already-detailed codebook drops accuracy sharply on theory-dependent tasks, so the model is not indifferent to content — only to surplus content. For researchers in other domains, the practical question is therefore not whether to invest time in prompt engineering, but whether to invest it in enriching the codebook itself — adding the category definitions, boundary cases, and worked examples that our codebooks already provide.

This raises a diagnostic question: how can a researcher tell whether their prompt is already well-specified or still underspecified? Our results suggest that robustness to surface variation is itself the signal: a well-specified prompt produces consistent classifications regardless of how it is formatted, framed, or paraphrased, while an underspecified prompt does not. Paraphrasing is one such surface variation, and it is exactly how [32] measure prompt stability. What their framework does not address is why one prompt is stable and another is not. Our results point to a candidate explanation: stability emerges once the prompt is saturated with the information the model can effectively use, after which further content or rewording yields only marginal gains. Practical tests for when a given prompt and model have reached this saturation point are a natural direction for future work.

### 7.3. Open or closed models

**Fig 3:**
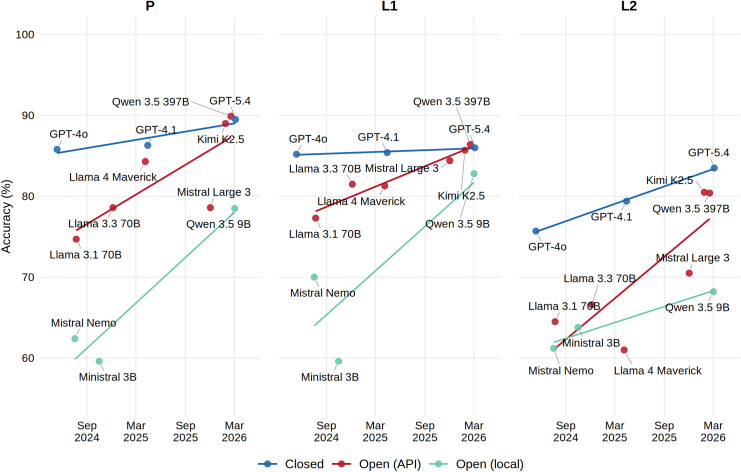
Model performance over time by task. Accuracy on verbatim *n*-shot prompts (with reasoning where available) across model generations. Blue = proprietary GPT family; red = frontier open-weight models; green = small open-weight models (≤10B) that can run on consumer hardware.

Fig 3 plots accuracy over time for models featured in the prompting literature we cite, alongside additional models we evaluated to broaden the comparison (Beyond the four models analysed in this paper, we ran the same verbatim *n*-shot classification on GPT 4o, GPT 4.1, Llama 3.1 70B, Llama 3.3 70B, Llama 4 Maverick, Kimi K2.5, Mistral Large 3, Mistral NeMo (12B), and Ministral 3B. These additional runs are used here for the timeline comparison only; the factorial experiments reported in the results are limited to the four featured models.). Three trends are visible. First, frontier open-weight models (red) have converged toward proprietary performance (blue) over two years, with the steepest gains on *L*-tasks: Qwen 3.5 397B now matches GPT 5.4 on *P* and *L*_I_. Second, small open-weight models that can run on consumer hardware (green, ≤10B parameters) are also improving rapidly — Qwen 3.5 9B reaches 82–84% on *L*_I_, a level that required frontier models a generation earlier. Third, GPT 5.4 retains a clear lead on the hardest task (*L*_II_), where the gap between proprietary and open models is still substantial.

A closing accuracy gap does not mean open models are equally dependable in practice. Smaller open-weight models remain more sensitive to surface variations: formatting, framing, and wording changes that have no effect on GPT 5.4 can produce significant shifts for Qwen 9B (Section 6.2). This matters for the distinction between *reproducibility* (repeating the same analysis with the same prompt and model) and *replicability* (arriving at the same conclusions with reasonable variations in prompt design). An open model sensitive to surface variations may reproduce well (the same prompt and model weights yield the same outputs) yet fail to generalise when another researcher adapts the prompt to a new setting with minor formatting, framing, or phrasing changes (No LLM classification is perfectly reproducible: even with identical prompts, temperature 0, and the same model, run-to-run variation produces flip rates of 2–12% depending on the model and task [41, 42]. We use “reproduce” here in the weaker sense of obtaining substantively similar results.). GPT 5.4’s insensitivity to these choices makes it more replicable, not just more accurate.

The main argument against proprietary models is reproducibility: if the provider deprecates the model, the exact same classification can no longer be reproduced. But how bad is this problem in practice? Inter-model agreement across three consecutive GPT generations (4o, 4.1, 5.4) on identical items is high (We measure inter-model agreement using Krippendorff’s α, a reliability coefficient that accounts for chance agreement; α=1 indicates perfect agreement, and values above α≈0.7 are commonly read as substantial agreement [32]. On our data, adjacent GPT generations reach α=0.89−0.91 on *P* and 0.84−0.88 on *L*_I_; the full table is in S7 Appendix.). A researcher who classified data with GPT 4o and later re-ran the analysis with GPT 5.4 would obtain higher accuracy, potentially greater robustness to prompt variation, and classification agreement comparable to substituting one trained human annotator for another (For reference, on the *P* task the two human annotators in our source data [2] reach Krippendorff’s α=0.48 before disagreements were resolved — considerably below the cross-generation GPT agreement of 0.89−−0.91 reported above.). This is not perfect reproducibility, but it is a predictable and improving trajectory — and no worse than the inter-coder reliability one would accept when replacing one human annotator with another [18].

Even our largest open-weight model, Qwen 3.5 397B, remains sensitive to surface-level prompt variation in a way that GPT 5.4 is not (Section 6.2). Reaching frontier-level robustness therefore appears to require a further scale jump for open models, plausibly to the trillion-parameter range that closed frontier models such as GPT 5.4 are believed to inhabit. On the open side, Moonshot’s Kimi K2.5 currently exemplifies that range. (Kimi K2.5 was the only other state-of-the-art open-weight model released as recently as the Qwen 3.5 series at the time of writing. We did not include it in our factorial experiments because its non-reasoning mode is forced to temperature 0.6, and we did not want a model whose outputs come from a temperature level other than 0 to be mixed with the rest of our temperature-0 runs.)

### 7.4. Limitations

Experimental-economics text is a best-case setting for codebook-based classification, and our results should be read with this in mind. Three features of our setting are unlikely to hold jointly in other social-science domains. Beyond the codebook quality discussed in Section 7.2, the text we classify is tightly constrained: subjects communicate within fixed experimental rules with known payoffs and structured protocols, producing a narrow vocabulary and a clear mapping between language and the concepts being classified. Tweets, parliamentary speeches, Reddit threads, or interview transcripts are far more variable in style, length, and ambiguity, and a model that ignores surface variations in our setting may not do so once the text itself becomes more variable. We are also able to provide the full experimental and theoretical context in the prompt because we designed the experiments; most applied settings lack this, leaving the researcher with a codebook and the text but no complete description of the data-generating process.

These conditions make our null finding on surface features a strong result within this setting; whether the same holds in domains where any of these conditions is relaxed remains an open question. The content-ablation results show what happens when prompt detail drops: accuracy on *L*-tasks falls from above 80% to below 50% when classification instructions are reduced to one line. Researchers working with codebooks closer to our minimal condition than our verbatim condition, as many social-science codebooks are, may find that the prompt design choices we show to be irrelevant here do matter for them. This can stem from two sources: their codebook may not yet give the model enough information to ignore how it is presented, or the concepts being classified may be intrinsically more ambiguous, limiting what any codebook can fully specify.

## 8. Conclusion

For frontier models on codebook-based classification tasks, the practical recommendation is clear: transcribe the codebook faithfully, include the worked examples it provides, and use a capable model. This yields 82–88% agreement with humans across the four models we test. Detailed classification instructions and worked examples are the two drivers of accuracy. The effect of surface variations on classification outcome becomes negligible as the level of informational detail of the prompt increases. Furthermore, model reasoning does not substitute for missing detail, adding little for capable models once detail is present.

These findings suggest a shift in attention from prompt engineering toward two factors that actually matter: the level of informational detail of the classification information provided to the model, and the choice of a model capable of processing that information reliably. The effort conventionally allocated to formatting, persona assignment, and iterative wording refinement is better invested in ensuring that the classification instructions are clear and detailed.

## Supporting information

S1 Appendix Content ablation. (PDF)

S2 Appendix Format and framing. (PDF)

S3 Appendix Examples. (PDF)

S4 Appendix Reasoning. (PDF)

S5 Appendix Information fidelity. (PDF)

S6 Appendix Full experiment designs. (PDF)

S7 Appendix Cross-generation agreement. (PDF)

S8 Appendix API and implementation details. (PDF)

S9 Appendix Cost and latency. (PDF)

S10 Appendix Codebook-to-prompt pipeline: prompts. (PDF)

S11 Appendix Prompt template rationale. (PDF)

S12 Appendix Prior work comparison. (PDF)

S13 Appendix F1 metric replication. (PDF)
