# Supplementary material for: Much Ado about Prompting: LLM classification of text messages from experiments
Source: PLoS One. 2026 Aug 4;21(8):e0354757. doi: 10.1371/journal.pone.0354757 (PMC13436748; doi:10.1371/journal.pone.0354757)
Supplement: Supplementary file 1 [file pone.0354757.s001.pdf]

S1 Appendix — Content Ablation

Supporting Information for "Much Ado about Prompting: LLM Classification of Text Messages from Experiments"

Can Çelebi   Stefan P. Penczynski

1 Content Ablation

1.1 Information surfaces

Figure **??** presents L_{II} information surfaces for all four models. Accuracy increases monotonically with both experiment context and theory context, and the gain from detailed classification instructions is visible as a shift between the left (simple) and right (verbatim) panels. GPT 5.4 and Qwen 397B show relatively flat surfaces on L_{I} (Figure 2), indicating robustness to context removal; GPT 5.4-nano and Qwen 9B show steeper gradients, reflecting greater dependence on prompt detail. On L_{II}, all models show steeper gradients, consistent with the greater role of theory context for this task.

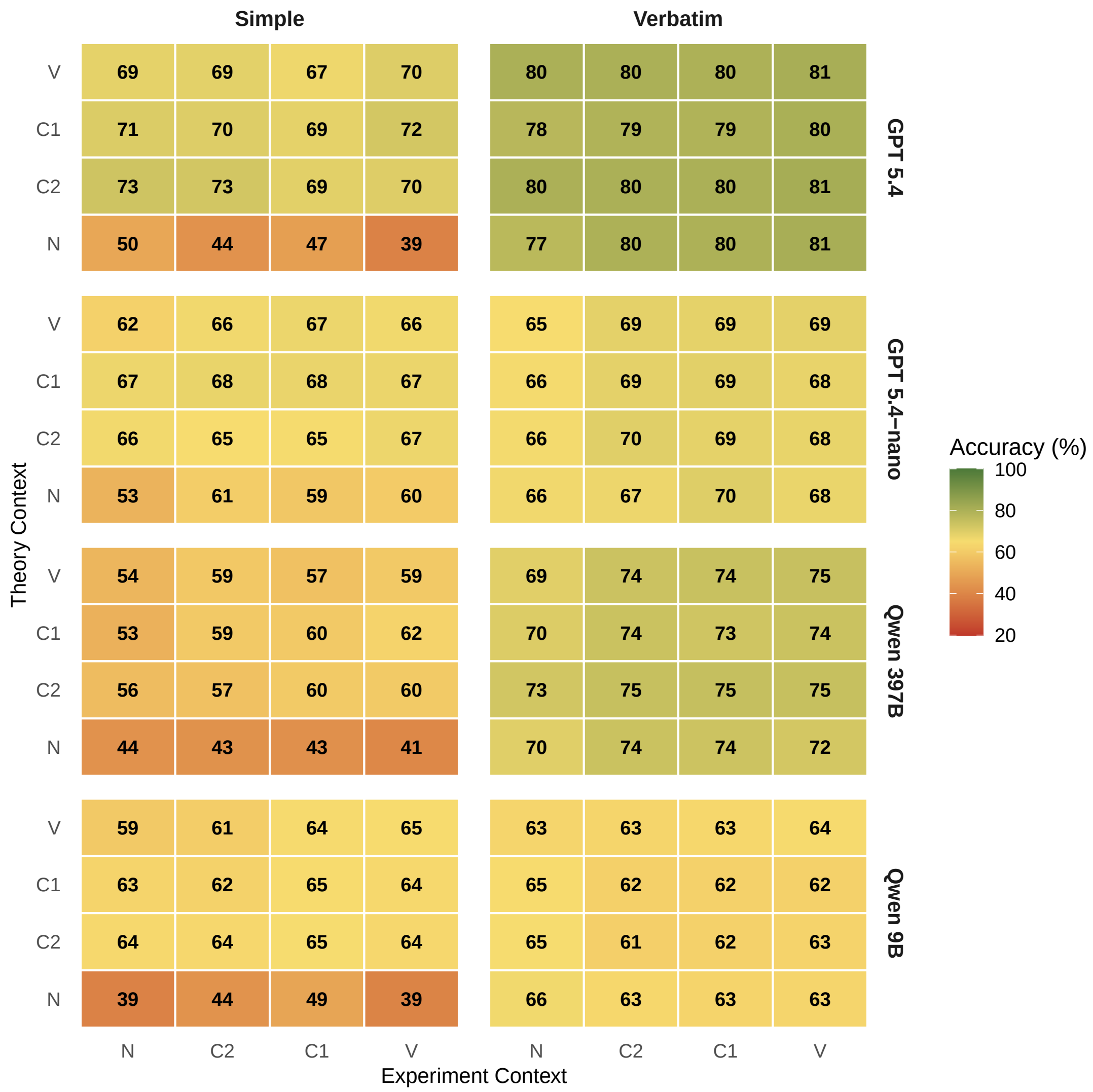

Figure 1: L_{II} information surface (all four models). Accuracy by experiment context (rows) and theory context (columns) under simple (left) and verbatim (right) classification instructions.

1.2 Variance decomposition

	P	L_{I}	L_{II}
Classification instructions	1.0%	58.3%	27.4%
Theory context	—	8.5%	9.6%
Experiment context	1.1%	1.3%	1.9%
Model	97.8%	31.9%	61.1%

Note: Unique explained variance, Type III likelihood-ratio tests. Each row: unique contribution after accounting for all others.

Table 1: Variance explained by component.

Table **??** reports the per-model variance decomposition using the same Type III likelihood-ratio methodology. The pattern confirms that model scale determines which prompt component dominates: for GPT 5.4, experiment context explains 81.5% of variance on P (classification instructions only 18.5%), whereas GPT 5.4-nano's variance on L_{I} is almost entirely driven by classification instructions (99.9%).

	P		L_{I}			L_{II}		
Model	Cls	Exp	Cls	Theory	Exp	Cls	Theory	Exp
GPT 5.4	18.5	81.5	57.4	41.6	0.9	69.7	30.2	0.1
GPT 5.4-nano	57.6	42.4	99.9	0.1	0.0	39.2	40.7	20.1
Qwen 397B	5.9	94.1	70.3	16.3	13.4	85.5	12.2	2.3
Qwen 9B	0.0	100.0	97.9	1.2	0.9	26.1	73.8	0.0

Note: Unique explained variance (%), Type III likelihood-ratio tests within each model. P has no theory dimension. Rows may not sum to 100 due to shared variance.

Table 2: Per-model variance decomposition (%).

1.3 Full cosine similarity table

Table **??** reports the cosine similarity between each compressed prompt variant and its verbatim source. For each text, we submit the full content as a single input to OpenAI's `text-embedding-3-large` model (via the Azure OpenAI API) and obtain a 3072-dimensional embedding vector; the reported similarity is the cosine of the angle between the verbatim and compressed vectors. Cosine similarity ranges from -1 (opposite) to 1 (identical), with 0 indicating orthogonal vectors; for embeddings of related natural-language texts, values above ~ 0.8 indicate that the two texts encode highly overlapping semantic content. Compression ratios show the fraction of verbatim word count retained.

Task	Source	Level	Cosine	Words	Compression
P	Exp. context	V	1.000	108	100%
P	Exp. context	C1	0.978	63	58%
P	Exp. context	C2	0.866	33	31%
P	Cls. instructions	V	1.000	—	100%
P	Cls. instructions	S	0.772	—	11%
L_{I}	Exp. context	V	1.000	293	100%
L_{I}	Exp. context	C1	0.857	174	59%
L_{I}	Exp. context	C2	0.831	97	33%
L_{I}	Theory	V	1.000	735	100%
L_{I}	Theory	C1	0.901	374	51%
L_{I}	Theory	C2	0.804	158	21%
L_{I}	Cls. instructions	V	1.000	—	100%
L_{I}	Cls. instructions	S	0.724	—	3%
L_{II}	Exp. context	V	1.000	732	100%
L_{II}	Exp. context	C1	0.971	362	49%
L_{II}	Exp. context	C2	0.940	226	31%
L_{II}	Theory	V	1.000	362	100%
L_{II}	Theory	C1	0.948	198	55%
L_{II}	Theory	C2	0.839	94	26%
L_{II}	Cls. instructions	V	1.000	—	100%
L_{II}	Cls. instructions	S	0.722	—	5%

Note: Minimal (N) removes the section entirely (0% compression). Classification instruction word counts omitted as they vary by task structure. Embeddings computed with Azure OpenAI `text-embedding-3-large` (3072 dimensions).

Table 3: Cosine similarity and compression ratio for all prompt variants.
