# Supplementary material for: Much Ado about Prompting: LLM classification of text messages from experiments
Source: PLoS One. 2026 Aug 4;21(8):e0354757. doi: 10.1371/journal.pone.0354757 (PMC13436748; doi:10.1371/journal.pone.0354757)
Supplement: Supplementary file 2 [file pone.0354757.s002.pdf]

S2 Appendix — Format and Framing

Supporting Information for "Much Ado about Prompting: LLM Classification of Text Messages from Experiments"

Can Çelebi Stefan P. Penczynski

1 Format and Framing

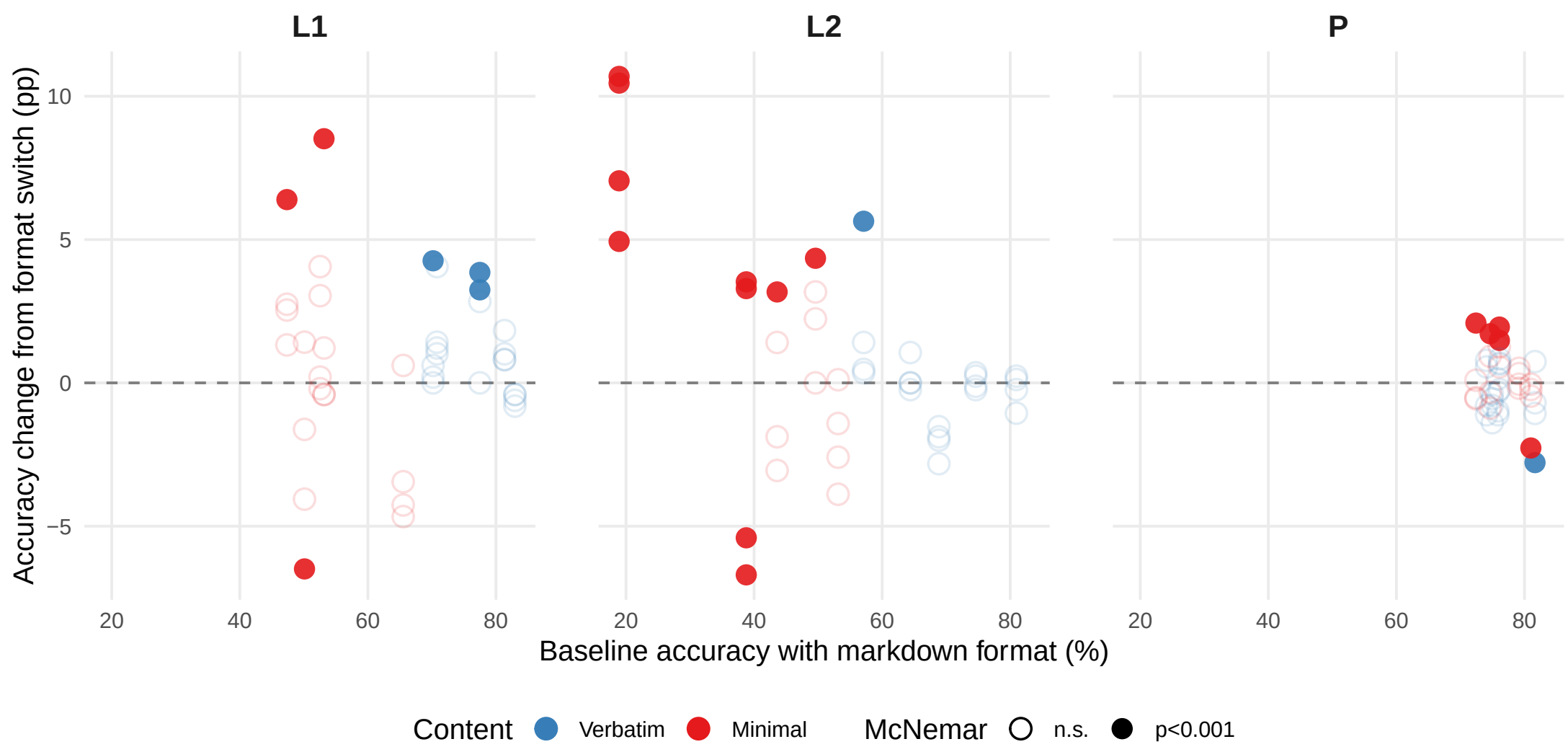

Figure 1: Format effects by task.

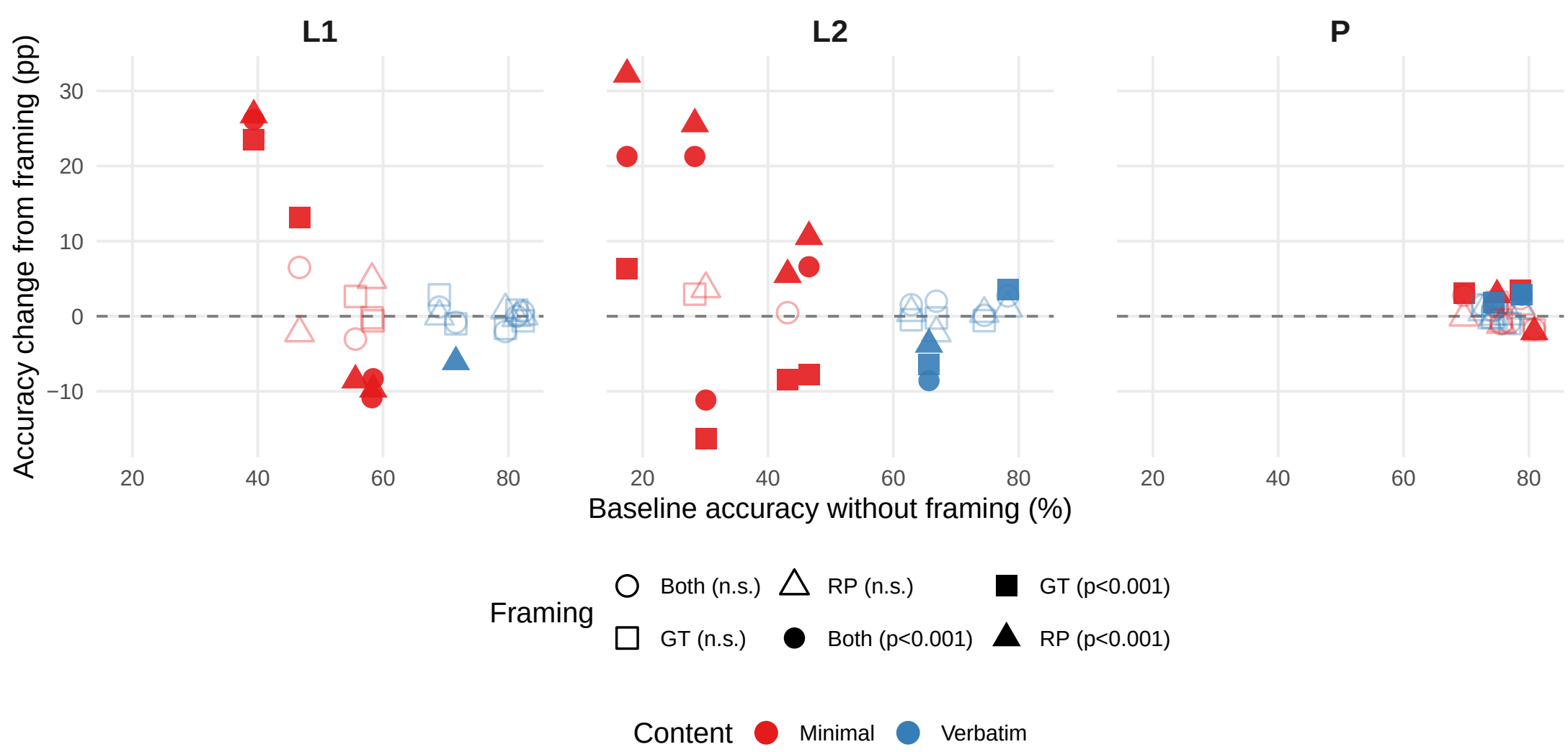

Figure 2: Framing effects by task.

Table **??** reports the mean accuracy change ($\bar{\Delta}$, pp) and its standard deviation (σ) across format and framing treatments relative to the baseline. Format cells pool 12 comparisons each (4 alternative formats $\times$ 3 tasks); framing cells pool 9 comparisons each (3 framing conditions $\times$ 3 tasks, vs. the no-framing baseline).

Table 1: Format and framing: mean $\bar{\Delta}$ (pp) and σ vs. baseline.

	Format				Framing			
	Minimal		Verbatim		Minimal		Verbatim	
Model	$\bar{\Delta}$	σ	$\bar{\Delta}$	σ	$\bar{\Delta}$	σ	$\bar{\Delta}$	σ
GPT 5.4	+1.5	2.5	−0.2	1.6	+6.0	10.9	+1.9	1.7
GPT 5.4-nano	+0.2	2.3	−0.6	1.8	+1.0	6.4	+0.4	1.5
Qwen 397B	−0.9	2.7	+0.1	0.7	−3.2	4.9	−0.3	0.7
Qwen 9B	+0.5	4.0	+0.6	1.4	+7.9	12.3	−0.2	2.5

1.1 Framing significance — all models

Table **??** extends the framing results (Table 4) by reporting the number of significant effects by framing treatment. On verbatim prompts, only GPT 5.4 shows any appreciable framing effects (3 of 9 comparisons, all positive); Qwen 9B has one significant negative effect, and the other two models show none. On minimal prompts, framing effects are more common but inconsistent in direction: GPT 5.4-nano and Qwen 397B both reach 5 of 9, but Qwen 397B's effects are predominantly negative (4 of 5) while Qwen 9B's are all positive (4 of 4).

	Verbatim				Minimal			
	Total	+	−	% neg	Total	+	−	% neg
GPT 5.4	3	3	0	0%	4	3	1	25%
GPT 5.4-nano	0	0	0	—	5	3	2	40%
Qwen 397B	0	0	0	—	5	1	4	80%
Qwen 9B	1	0	1	100%	4	4	0	0%

Note: Number of McNemar comparisons reaching $p < 0.001$ out of 9 per cell (3 framing treatments $\times$ 3 tasks, vs. no-framing baseline). +/− = direction of significant effect.

Table 2: Framing: number of significant effects by model and content level.

Figure **??** shows per-model framing effects at both content levels. Each point represents one task $\times$ framing treatment comparison against the no-framing baseline; filled shapes indicate significance at $p < 0.001$. Error bars show ± 1 standard deviation of the effects within each content level.

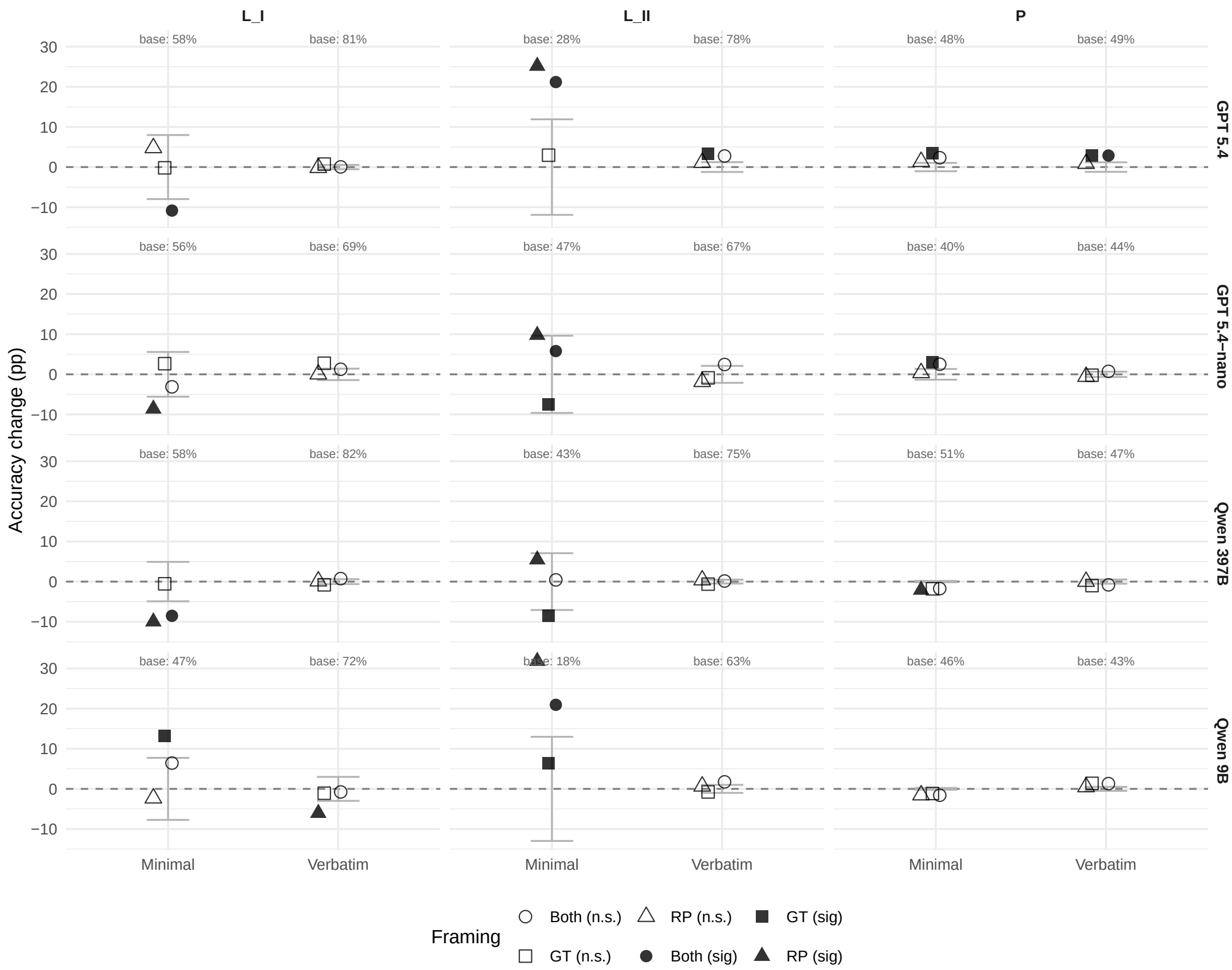

Figure 3: Per-model framing effects. Shapes encode framing type (GT only, RP only, Both) and significance. Baseline accuracy annotated at the top of each panel.
