# Supplementary material for: Much Ado about Prompting: LLM classification of text messages from experiments
Source: PLoS One. 2026 Aug 4;21(8):e0354757. doi: 10.1371/journal.pone.0354757 (PMC13436748; doi:10.1371/journal.pone.0354757)
Supplement: Supplementary file 3 [file pone.0354757.s003.pdf]

S3 Appendix — Examples

Supporting Information for "Much Ado about Prompting:
LLM Classification of Text Messages from Experiments"

Can Çelebi Stefan P. Penczynski

1 Examples

1.1 Example counts by label

Task	Label	Examples
P	Promise (1)	11
P	Empty talk (0)	10
L_{I}	Level 0	3
L_{I}	Level 1	2
L_{I}	Level 2	6
L_{I}	Level 3	2
L_{II}	Level 0	2
L_{II}	Level 1	2
L_{II}	Level 2	1
L_{II}	Level 3	0

Note: Counts reflect the worked examples available in each codebook. P: 21 total (10.5 per label). L_{I}: 13 total (3.25 per level). L_{II}: 5 total (1.25 per level); level 3 is unrepresented.

Table 1: Number of worked examples per classification label.

1.2 Content–examples substitution (all models)

Tables **??**–**??** report the same decomposition as Table 5 for the remaining three models.

	P	L_{I}	L_{II}
Δ_{Content}	$+3.3$	$+17.7^{*}$	$+15.8^{*}$
Δ_{Ex}	$+9.1^{*}$	$+20.1^{*}$	$+10.8^{*}$
$\Delta_{\text{Ex}\|\text{Content}}$	$+7.7^{*}$	$+8.1^{*}$	$+1.8$
$\Delta_{\text{Content}\|\text{Ex}}$	$+1.9$	$+5.7$	$+6.8^{*}$

Table 2: GPT 5.4-nano: content–examples decomposition.

	P	L_{I}	L_{II}
Δ_{Content}	-4.3^{*}	$+32.9^{*}$	$+31.0^{*}$
Δ_{Ex}	$+16.5^{*}$	$+32.1^{*}$	$+25.3^{*}$
$\Delta_{\text{Ex\|Content}}$	$+20.5^{*}$	$+0.6$	$+3.2$
$\Delta_{\text{Content\|Ex}}$	-0.3	$+1.4$	$+8.9^{*}$

Table 3: Qwen 397B: content–examples decomposition.

	P	L_{I}	L_{II}
Δ_{Content}	-0.9	$+17.7^{*}$	$+25.6^{*}$
Δ_{Ex}	$+14.2^{*}$	$+12.4^{*}$	$+30.6^{*}$
$\Delta_{\text{Ex\|Content}}$	$+15.3^{*}$	$+12.0^{*}$	$+3.8$
$\Delta_{\text{Content\|Ex}}$	$+0.2$	$+17.3^{*}$	-1.2

Table 4: Qwen 9B: content–examples decomposition.

1.3 Incremental effects — all models

Tables **??** and **??** report the accuracy at four key conditions for all four models: minimal 0-shot (baseline), minimal n-shot (+examples), verbatim 0-shot (+content), and verbatim n-shot (+both).

	Minimal		Verbatim	
	0-shot	n-shot	0-shot	n-shot
GPT 5.4	73.2	89.8	74.2	88.2
GPT 5.4-nano	60.9	70.0	64.3	72.0
Qwen 397B	70.5	87.0	66.2	86.7
Qwen 9B	64.1	78.3	63.3	78.5

Note: No reasoning. Wilson 95% CIs omitted for space.

Table 5: P: Accuracy (%) at four key conditions, all models.

	L_{I}				L_{II}			
	Minimal		Verbatim		Minimal		Verbatim	
	0-shot	n-shot	0-shot	n-shot	0-shot	n-shot	0-shot	n-shot
GPT 5.4	47.4	84.8	81.3	84.0	49.6	70.0	81.0	81.7
GPT 5.4-nano	52.5	72.6	70.2	78.3	53.1	63.9	68.9	70.7
Qwen 397B	50.1	82.2	83.0	83.6	43.6	68.9	74.6	77.8
Qwen 9B	53.1	65.5	70.8	82.8	38.8	69.4	64.4	68.2

Note: No reasoning. Wilson 95% CIs omitted for space.

Table 6: L_{I} and L_{II}: Accuracy (%) at four key conditions, all models.

1.4 Example Format

As with prompt formatting (Section 6.2), we also tested whether how examples are presented within the prompt matters. We compared five presentation styles — markdown headers, XML tags, plain delimiters, arrow notation, and multi-turn conversation — at both content levels. In the first four styles, examples are embedded in the system message alongside the codebook content. In the multi-turn style, each example is sent as a separate user–assistant exchange before the classification query.

Table **??** reports the proportion of significant effects when switching away from the markdown baseline to alternative example formats.

	In-prompt formats				Multi-turn			
	Minimal		Verbatim		Minimal		Verbatim	
	+	−	+	−	+	−	+	−
GPT 5.4	.00	.00	.00	.00	.00	.33	.00	.33
GPT 5.4-nano	.00	.00	.00	.00	.00	.67	.00	.00
Qwen 397B	.00	.33	.00	.33	.00	.67	.00	.33
Qwen 9B	.22	.33	.00	.33	.00	.67	.00	.33

Note: Proportion of all comparisons that are significantly positive (+) or negative (−) at $p < 0.001$, vs. markdown baseline. In-prompt: 9 per cell (3 formats × 3 tasks). Multi-turn: 3 per cell (1 format × 3 tasks).

Table 7: Example format: proportion of significant effects.

For GPT models, in-prompt format variation produces zero significant effects; only multi-turn is harmful. Qwen models are more sensitive: even in-prompt format switches produce up to .33 significant negatives, and multi-turn adds further damage (.67 on minimal). Overall, switching between in-prompt styles produces no significant effect for GPT models and only scattered effects for Qwen models. The one exception is

multi-turn presentation: placing examples outside the system message as conversation history consistently hurts all models, particularly on minimal prompts.

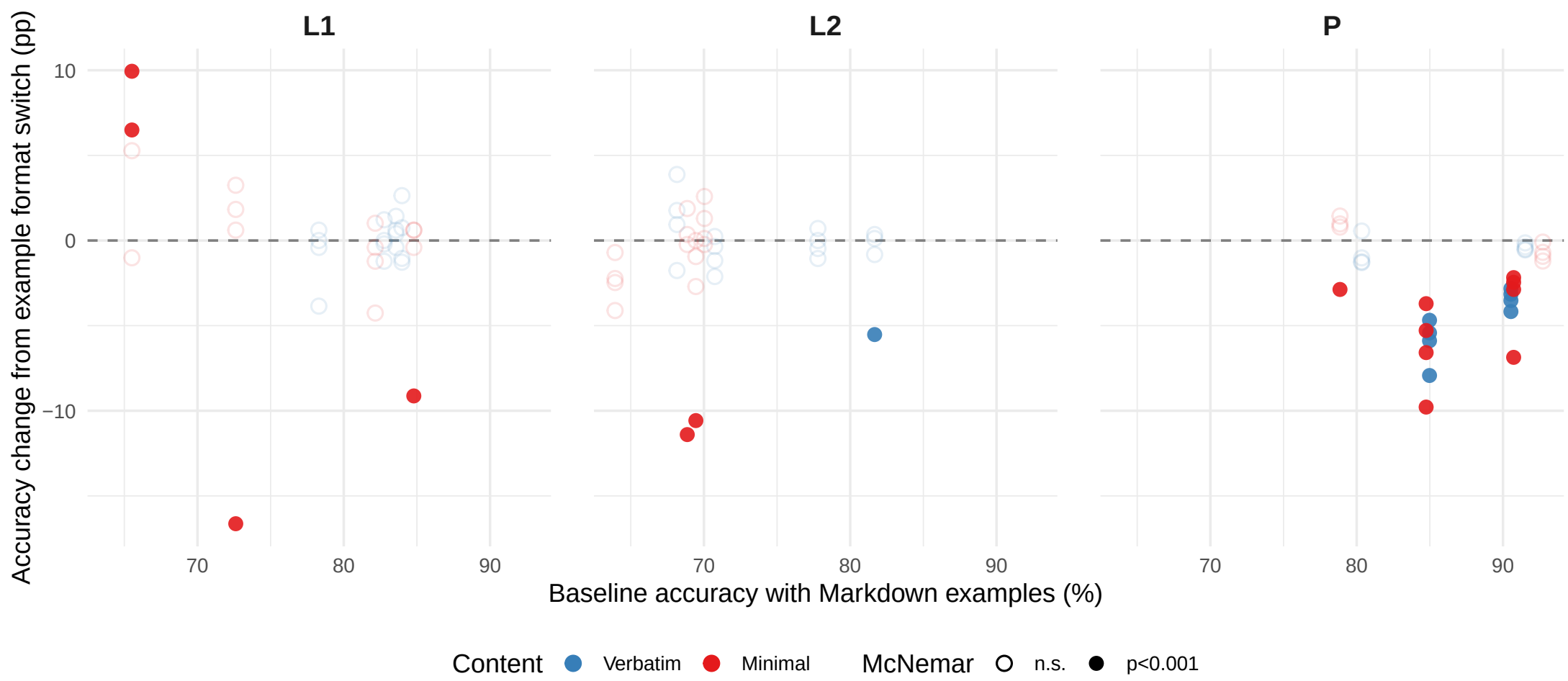

Figure 1: Example format effects by task.

Table **??** summarises the mean accuracy change ($\bar{\Delta}$, pp) and its standard deviation (σ) when switching away from the markdown baseline to alternative example formats. In-prompt formats (XML, delimiter, arrow) pool 9 comparisons per cell (3 alternative formats $\times$ 3 tasks); multi-turn pools 3 comparisons (1 format $\times$ 3 tasks).

Table 8: Example format: mean $\bar{\Delta}$ (pp) and σ vs. markdown baseline.

	In-prompt (9)				Multi-turn (3)			
	Minimal		Verbatim		Minimal		Verbatim	
Model	$\bar{\Delta}$	σ	$\bar{\Delta}$	σ	$\bar{\Delta}$	σ	$\bar{\Delta}$	σ
GPT 5.4	−0.2	1.0	−0.4	0.6	−2.2	6.2	−1.2	4.1
GPT 5.4-nano	+0.2	2.5	−0.4	0.9	−7.1	8.4	−2.6	1.1
Qwen 397B	−1.0	2.1	−1.3	2.5	−8.5	3.7	−2.2	3.3
Qwen 9B	−1.2	6.3	−2.4	4.1	−6.4	10.3	−2.5	7.7
