# Supplementary material for: Much Ado about Prompting: LLM classification of text messages from experiments
Source: PLoS One. 2026 Aug 4;21(8):e0354757. doi: 10.1371/journal.pone.0354757 (PMC13436748; doi:10.1371/journal.pone.0354757)
Supplement: Supplementary file 4 [file pone.0354757.s004.pdf]

S4 Appendix — Reasoning

Supporting Information for "Much Ado about Prompting: LLM Classification of Text Messages from Experiments"

Can Çelebi Stefan P. Penczynski

1 Reasoning

1.1 Reasoning dosage — GPT models

Table **??** reports the incremental effect of each reasoning step on verbatim content for GPT 5.4 and GPT 5.4-nano. For GPT 5.4, the first step (off → low) produces significant gains at 0-shot, but every subsequent step is non-significant or significantly negative. At n-shot, even the first step is marginal. GPT 5.4-nano benefits more from the initial step, and shows significant gains from extra-high reasoning on L_{II} at both shot conditions — consistent with smaller models extracting more from extended reasoning.

		0-shot			n-shot		
	Step	P	L_{I}	L_{II}	P	L_{I}	L_{II}
GPT 5.4	off → low	+4.6*	+1.6	−1.9	+0.8	+0.6	−2.0
	low → med	−0.1	+1.0	+2.4	+0.5	+2.0	+1.9
	med → high	−5.7*	−1.4	−0.5	+0.0	−0.6	+2.0
	high → xhigh	−5.6*	+0.0	+0.7	−0.3	−1.4	−0.7
GPT 5.4-nano	off → low	+5.2*	+1.6	−1.1	+5.8*	+0.6	−4.1*
	low → med	+1.5	+5.1	+0.2	+4.1*	+0.6	+3.4
	med → high	−0.2	−1.2	+2.5	+1.1	−0.2	+0.4
	high → xhigh	−2.0	+4.7	+6.1*	+2.9*	+3.7	+8.3*

Note: * = McNemar $p < 0.001$. All values in pp, each step relative to the preceding level. Reasoning token counts: low ≈113, med ≈309, high ≈682, xhigh ≈2,479. Table **??** reports high reasoning to match Qwen's reasoning token output (≈750 tokens).

Table 1: Reasoning dosage: incremental step effects on verbatim content.

1.2 Effect of reasoning on verbatim content

Table **??** reports the effect of enabling reasoning on verbatim content at both zero-shot and n-shot for all four models.

	0-shot			n-shot		
	P	L_{I}	L_{II}	P	L_{I}	L_{II}
GPT 5.4	-1.1	$+1.2$	$+0.0$	$+1.3$	$+2.0$	$+1.9$
GPT 5.4-nano	$+6.5^{*}$	$+5.5$	$+1.6$	$+11.1^{*}$	$+1.0$	-0.4
Qwen 397B	$+8.4^{*}$	$+1.0$	$+0.6$	$+3.1^{*}$	$+2.8$	$+2.6$
Qwen 9B	$+9.4^{*}$	$+10.5^{*}$	$+2.4$	$+8.7^{*}$	$+1.6$	$+4.0$

Note: * = McNemar $p < 0.001$. All values in pp: accuracy with reasoning minus accuracy without, on verbatim content. GPT: high reasoning (≈682 tokens); Qwen: reasoning on (≈780 tokens).

Table 2: Effect of reasoning on verbatim content.

GPT 5.4 shows no significant effect on any task at any condition. Smaller models benefit selectively: on P, reasoning helps all three non-frontier models significantly at both 0-shot and n-shot, with the largest gains for Qwen 9B ($+9.4$ and $+8.7$ pp). On L_{I}, Qwen 9B gains $+10.5$ pp at zero-shot, but this drops to $+1.6$ pp (n.s.) once examples are present. On L_{II}, no model shows a significant reasoning effect at either condition.

1.3 Content vs. reasoning substitutability (all models)

Tables **??**–**??** extend Table 7 to the remaining three models. The pattern is consistent: content retains its full value when reasoning is active ($\Delta_{\text{Content}|\text{Rea}}$ is large on L-tasks), while reasoning given content ($\Delta_{\text{Rea}|\text{Content}}$) is near-zero on L-tasks for all models except Qwen 9B on L_{I}. On P, smaller models show significant reasoning-given-content effects, consistent with reasoning partially compensating for weaker pre-training representations.

	P	L_{I}	L_{II}
Δ_{Rea}	$+9.7^{*}$	$+11.2^{*}$	-9.5^{*}
$\Delta_{\text{Rea}} - \Delta_{\text{Content}}$	$+6.4^{*}$	-6.5	-25.3^{*}
$\Delta_{\text{Content}|\text{Rea}}$	$+0.1$	$+12.0^{*}$	$+26.9^{*}$
$\Delta_{\text{Rea}|\text{Content}}$	$+6.5^{*}$	$+5.5$	$+1.6$

Table 3: GPT 5.4-nano: content vs. reasoning substitutability.

	P	L_{I}	L_{II}
Δ_{Rea}	$+0.1$	$+5.3$	-1.6
$\Delta_{\text{Rea}} - \Delta_{\text{Content}}$	$+4.4^{*}$	-27.6^{*}	-32.7^{*}
$\Delta_{\text{Content}\mid\text{Rea}}$	$+3.9^{*}$	$+28.6^{*}$	$+33.2^{*}$
$\Delta_{\text{Rea}\mid\text{Content}}$	$+8.4^{*}$	$+1.0$	$+0.6$

Table 4: Qwen 397B: content vs. reasoning substitutability.

	P	L_{I}	L_{II}
Δ_{Rea}	$+2.9$	$+5.4$	-11.6^{*}
$\Delta_{\text{Rea}} - \Delta_{\text{Content}}$	$+3.7$	-12.3^{*}	-37.2^{*}
$\Delta_{\text{Content}\mid\text{Rea}}$	$+5.7^{*}$	$+22.8^{*}$	$+39.6^{*}$
$\Delta_{\text{Rea}\mid\text{Content}}$	$+9.4^{*}$	$+10.5^{*}$	$+2.4$

Table 5: Qwen 9B: content vs. reasoning substitutability.
