# Supplementary material for: Much Ado about Prompting: LLM classification of text messages from experiments
Source: PLoS One. 2026 Aug 4;21(8):e0354757. doi: 10.1371/journal.pone.0354757 (PMC13436748; doi:10.1371/journal.pone.0354757)
Supplement: Supplementary file 5 [file pone.0354757.s005.pdf]

S5 Appendix — Information Fidelity

Supporting Information for "Much Ado about Prompting:
LLM Classification of Text Messages from Experiments"

Can Çelebi Stefan P. Penczynski

1 Information Fidelity

1.1 Verbosity and noise

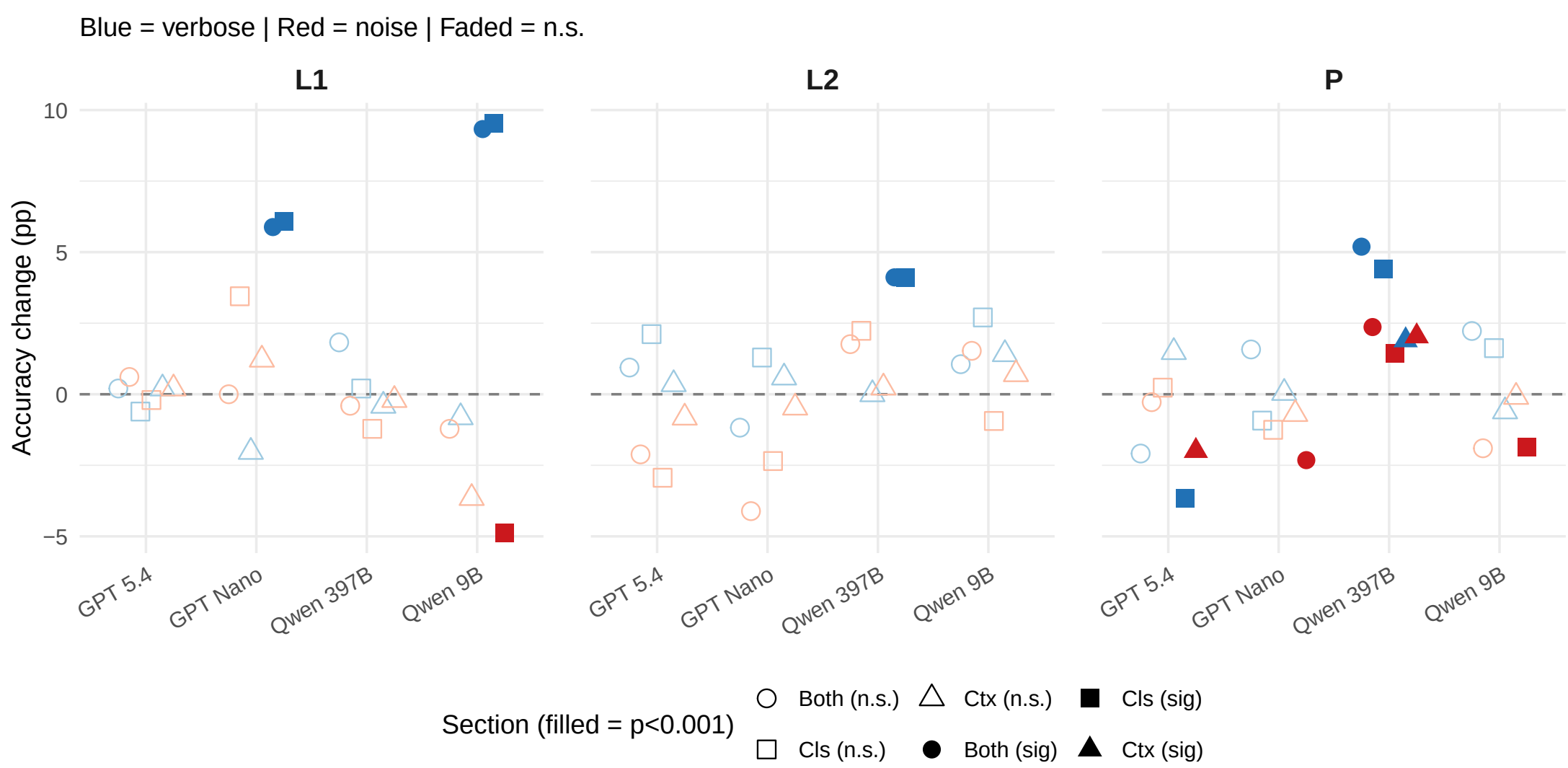

Figure 1: Verbosity and noise effects (0-shot).

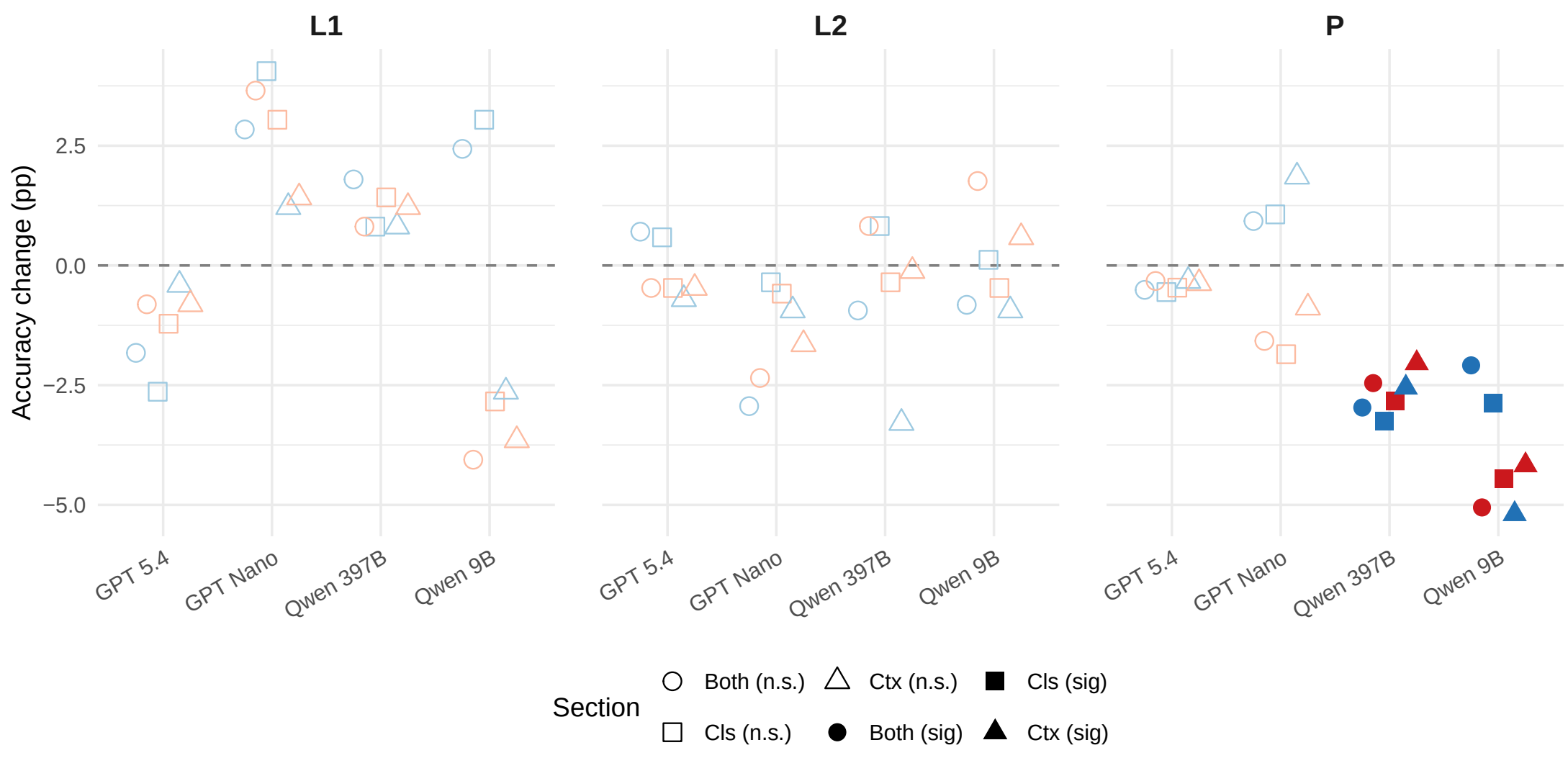

Figure 2: Verbosity and noise effects (n-shot).

Table **??** reports the mean accuracy change ($\bar{\Delta}$, pp) and its standard deviation (σ) for verbosity and noise treatments relative to the baseline. Each cell pools 9 comparisons (3 levels $\times$ 3 tasks).

Table 1: Verbosity and noise: mean $\bar{\Delta}$ (pp) and σ vs. baseline.

	0-shot				n-shot			
	Verbose		Noise		Verbose		Noise	
Model	$\bar{\Delta}$	σ	$\bar{\Delta}$	σ	$\bar{\Delta}$	σ	$\bar{\Delta}$	σ
GPT 5.4	−0.3	2.4	−0.9	1.4	−0.7	1.0	−0.7	0.3
GPT 5.4-nano	+1.3	3.0	−0.9	2.3	+1.1	2.2	−0.2	2.4
Qwen 397B	+2.9	2.8	+1.2	1.6	−1.4	2.6	−0.7	2.2
Qwen 9B	+3.1	3.8	−1.5	2.1	−1.5	3.2	−3.1	3.1

1.2 Paraphrasing

Each content section of the verbatim prompt is independently translated to a target language (Turkish or German) and back to English using an LLM, producing a semantically equivalent text with altered wording and syntax. Cosine similarity to the original remains 0.97–0.99; token counts change by $\pm 5\%$. This makes wording the primary source of variation. Each language pair is tested on all 3 tasks at both 0-shot and n-shot, yielding 6 comparisons per model per shot condition (24 total per model).

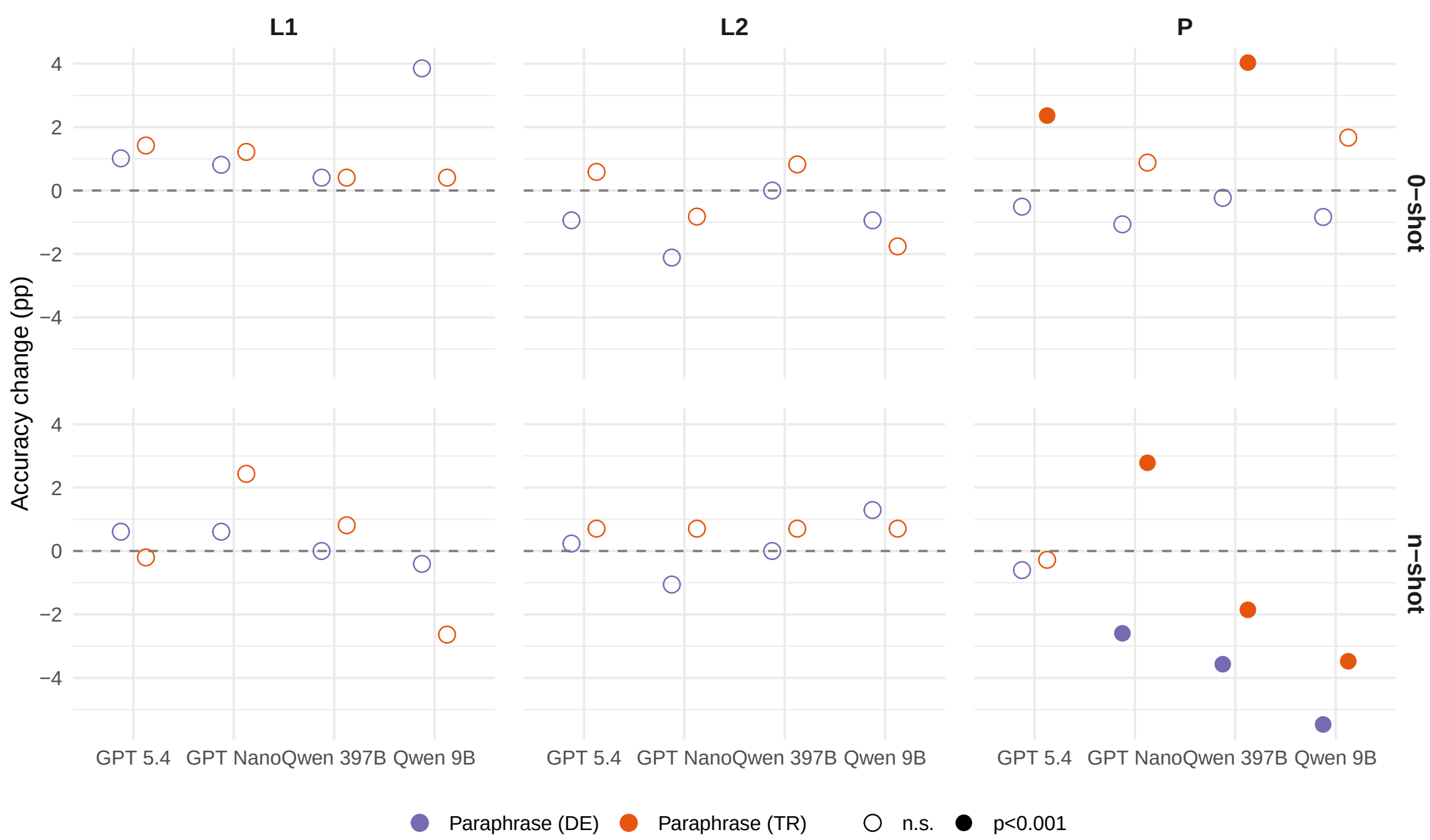

Figure 3: Paraphrase effects by task and model. Top: 0-shot. Bottom: n-shot. Orange = Turkish back-translation; purple = German back-translation. Filled = $p < 0.001$.

At 0-shot (Figure **??**, top), effects are negligible: the median absolute change is below 1 pp for all models, and GPT 5.4 shows zero significant effects across all 6 comparisons. L_{II} is entirely unaffected for every model. The direction of non-significant changes is evenly split — positive and negative with no pattern.

At n-shot (Figure **??**, bottom), a few significant effects emerge, concentrated on P for smaller models. GPT 5.4 remains unaffected. The significant n-shot effects are predominantly negative, suggesting that paraphrasing occasionally disrupts the alignment between example wording and classification criteria — but only for models that are already sensitive to surface variation, and only when examples are present to create a wording mismatch.
