# Supplementary material for: Much Ado about Prompting: LLM classification of text messages from experiments
Source: PLoS One. 2026 Aug 4;21(8):e0354757. doi: 10.1371/journal.pone.0354757 (PMC13436748; doi:10.1371/journal.pone.0354757)
Supplement: Supplementary file 6 [file pone.0354757.s006.pdf]

S6 Appendix — Full Experiment Designs

Supporting Information for "Much Ado about Prompting: LLM Classification of Text Messages from Experiments"

Can Çelebi   Stefan P. Penczynski

1 Full Experiment Designs

1.1 Content Ablation

Each prompt is assembled from three codebook sections: experiment context, theory context (L-tasks only), and classification instructions. Each section is varied independently across compression levels:

Section	Levels	Description
Experiment context	V, C1, C2, N	Verbatim, ≈50%, ≈25%, removed
Theory context	V, C1, C2, N	Verbatim, ≈50%, ≈25%, removed
Classification instructions	V, S	Verbatim, simplified (1-line)

P-tasks have no theory dimension, yielding $4 \times 2 = 8$ variants per task. L-tasks cross all three dimensions: $4 \times 4 \times 2 = 32$ variants per task. Total: 80 variants per model (8 + 8 + 32 + 32).

Compressions are generated iteratively (V $\rightarrow$ C1, C1 $\rightarrow$ C2) using the structured compression prompt in Appendix **??**, which instructs the model to only remove content while preserving a task-specific list of key technical terms. Cosine similarity to the verbatim text remains above 0.80 at all levels (Table 2; full results for all tasks in Appendix 1.3). The filtering prompt used to prepare the verbatim text from the raw codebook is in Appendix **??**. Figure **??** illustrates the result on the L_{I} experiment context.

Verbatim (V) — 293 words

- A single experimental session consists of 4 parts and every part consists of 2 periods. In every part, every subject is randomly paired with another one to form a team.
- Depending on the treatment, every team then is randomly matched with 2 or 5 other teams to form a voting group.
- Every group will be assigned to a blue or red urn and every team in the group will draw a ball from their assigned urn. The blue urn contains twice more blue balls than red balls. The red urn contains twice more red balls than blue balls.
- [... five further statements: group size, draw mechanism, voting rule, message protocol, and final decision.]

C1 (≈50%) — 174 words

- Every subject is randomly paired with another one to form a team. Every team is randomly matched with other teams to form a voting group.
- Every group will be assigned to a blue or red urn and every team in the group will draw a ball from their assigned urn. The blue urn contains twice more blue balls than red balls. The red urn contains twice more red balls than blue balls.
- The groups consist of either 3 or 6 teams. The balls will be drawn from the urn with or without replacement.
- [...]

C2 (≈25%) — 97 words

- Subjects form teams of two. Teams are grouped into voting groups of 3 or 6 teams.
- Every group is assigned to a blue or red urn. The blue urn contains twice more blue balls than red balls, and vice versa. Every team draws a ball, with or without replacement.
- The goal is to correctly state the color of the urn. Every team submits a vote (red or blue); if every team votes red, the group decision is red, otherwise blue.
- Each subject sends a message to their team member explaining their suggested vote.

Figure 1: The L_{I} experiment context at three compression levels. Each level is produced from the previous one with the compression prompt in Appendix **??**. The C2 statements are given in full; V and C1 are truncated for space.

1.2 Format

Five visual formats decomposed into three binary features (markdown markers, section titles, bullet lists):

Format	Md markers	Titles	Bullets	Description
ml (baseline)	✓	✓	✓	`# headers` + `- bullets`
tl		✓	✓	Plain titles + `- bullets`
mp		✓		Plain titles + prose paragraphs
fl			✓	No headers + `- bullets`
pt				No headers + prose paragraphs

Each format is tested at two content levels (verbatim and minimal), yielding $4 \times 2 = 8$ new variants per task (the ml baseline is shared with Experiment A).

The following example shows the same classification instruction content rendered in each format:

```
ml (markdown + list)
## Level 0
- Chooses randomly, without justification or through some
justification completely unrelated to the task.
- Might not have understood the game or shows no interest in it.

tl (titles + list)
Level 0
- Chooses randomly, without justification or through some
justification completely unrelated to the task.
- Might not have understood the game or shows no interest in it.

mp (titles + prose)
Level 0
Chooses randomly, without justification or through some
justification completely unrelated to the task.  Might not have
understood the game or shows no interest in it.

fl (flat list)
[header removed]
- Chooses randomly, without justification or through some
justification completely unrelated to the task.
- Might not have understood the game or shows no interest in it.

pt (plain text)
[header removed]
Chooses randomly, without justification or through some
justification completely unrelated to the task.  Might not have
understood the game or shows no interest in it.
```

Figure 2: The same classification instruction in all five format treatments.

1.3 Framing

Two framing elements are tested by removal:

Condition	Role persona	General task
full (baseline)	✓	✓
norp		✓
nogt	✓	
none		

Each condition is tested at two content levels (verbatim and minimal), yielding $3 \times 2 = 6$ new variants per task (the "full" baseline is shared with Experiment A). Total: 12 files per model.

The "General Task" and "Role Persona" are the two framing sections of the prompt template (Figure 1); all other sections are held fixed. Figure **??** instantiates them for

L_I and shows the four framing conditions, following the template's markdown layout.

full (baseline) — general task + role persona

```
# General Task
- Classify a player's level of strategic thinking (0--3) based on
their message in a voting game experiment.

# Role Persona
- Act as a behavioral economist with expertise in text
classification, bounded rationality, and level-k reasoning.

# Context
- ...
```

norp — general task only

```
# General Task
- Classify a player's level of strategic thinking (0--3) based on
their message in a voting game experiment.
# Context
- ...
```

nogt — role persona only

```
# Role Persona
- Act as a behavioral economist with expertise in text
classification, bounded rationality, and level-k reasoning.
# Context
- ...
```

Figure 3: The L_I prompt under each framing condition, following the template in Figure 1. Only the # General Task and # Role Persona sections are added or removed; all other sections are held fixed.

1.4 Examples and Reasoning

Examples. Five example presentation styles are tested:

Style	Description
n (markdown)	Examples formatted with markdown headers and bullets
nx (XML)	Examples wrapped in `<example>` tags
nd (delimiter)	Examples separated by `---` delimiters
na (arrow)	Input → Output arrow format
t (multi-turn)	Examples as user/assistant conversation turns

Each style is crossed with 2 content levels × 3 tasks (P excluded — no worked examples in codebook) = 20 files per model.

The following example shows the same worked example (from the P task) rendered in each in-prompt style:

n (markdown)

```
## Example 1
### Message
P1: all 200 then?
P2: yes
### Classification
{``p1'':``0'', ...}
```

nx (XML)

```
<example>
  <message>
  P1: all 200 then?
  P2: yes
  </message>
  <classification>
  {``p1'':``0'', ...}
  </classification>
</example>
```

nd (delimiter)

```
Example 1:
Message: P1: all 200 then?
P2: yes
Classification: {``p1'':``0'', ...}
```

na (arrow)

```
``P1: all 200 then? P2: yes'' → {``p1'':``0'', ...}
```

t (multi-turn) --- *not in system prompt; sent as conversation turns:*

User: `P1: all 200 then? P2: yes`
Assistant: `{``p1'':``0'', ...}`

Figure 4: The same worked example in all five presentation styles.

Reasoning. GPT models support 4 reasoning effort levels (low, medium, high, extra-high); Qwen models support a binary toggle (off/on). Reasoning is crossed with content (verbatim/minimal) and shot (0-shot/n-shot), yielding up to 32 additional variants per model for GPT and 8 for Qwen.

1.5 Information Fidelity

Three manipulations test whether LLMs respond to informational content or textual surface.

Verbosity and noise. The verbatim prompt sections are either expanded with relevant elaboration (*verbose*: $+50$–275% words) or padded with irrelevant text of matched length (*noise*: red squirrel ecology facts interspersed between original sentences). Each manipulation is applied to three scopes, yielding a 2×3 design:

	Verbose		Noise	
Condition	Context	Cls	Context	Cls
cls only		✓		✓
ctx only	✓		✓	
both	✓	✓	✓	✓

Each of the 6 conditions (3 verbose + 3 noise) is tested at 0-shot and n-shot, yielding 12 variants per task. Unmarked sections retain their verbatim content. The noise control isolates the effect of prompt length from informational content: if verbosity helps because of the *content* added, then matched-length noise should not.

Paraphrasing. Round-trip back-translation (English → Turkish → English, English → German → English) preserves semantic content while changing surface wording. Tested at 0-shot and n-shot (14 files per model).

Raw codebook. The unprocessed codebook (with payment instructions, software notes, and formatting artifacts) is used directly as the prompt (1 file per task).

The following example shows the same experiment context passage in all three versions (from the P task):

```
Normal (verbatim)
- Participants can invest between 0--200 in a group project.
- Participants can keep any money that they don't invest.  The
total amount invested by all 3 group members is doubled.
- Then it is shared out equally.

Verbose (relevant elaboration, +50--275% words)
- Participants can invest between 0--200 in a group project.
This is a continuous choice --- participants can invest any
integer amount within this range, not just all-or-nothing.
- Participants can keep any money that they don't invest.  The
total amount invested by all 3 group members is doubled.  Then
it is shared out equally.  This means that from the individual
investor's perspective, investing 100 pence returns only 66
pence to the investor --- a net loss of 34 pence.  However, the
group collectively gains 200 pence (66 × 3) from that 100 pence
investment.

Noise (irrelevant padding, matched word count)
- Participants can invest between 0--200 in a group project.
- An adult red squirrel weighs between 250--340 grams and has a
body length of 19--23 cm plus a tail of 15--20 cm.
- Participants can keep any money that they don't invest.  The
total amount invested by all 3 group members is doubled.
- Red squirrels build nests called dreys, typically located 6--12
meters above ground, using twigs, moss, and stripped bark.
- Then it is shared out equally.
```

Figure 5: The same experiment context in three versions: normal, verbose (relevant elaboration), and noise (irrelevant padding matched in word count).
