# Supplementary material for: Much Ado about Prompting: LLM classification of text messages from experiments
Source: PLoS One. 2026 Aug 4;21(8):e0354757. doi: 10.1371/journal.pone.0354757 (PMC13436748; doi:10.1371/journal.pone.0354757)
Supplement: Supplementary file 7 [file pone.0354757.s007.pdf]

S7 Appendix — Cross-Generation Agreement

Supporting Information for "Much Ado about Prompting: LLM Classification of Text Messages from Experiments"

Can Çelebi Stefan P. Penczynski

1 Cross-Generation Agreement

Table ?? reports Krippendorff's α across three GPT generations (4o, 4.1, 5.4) on identical items using the verbatim n-shot prompt, treating each generation as a rater.

	P	L_{I}	L_{II}
4o – 4.1	.91	.88	.71
4.1 – 5.4	.89	.84	.66
4o – 5.4	.87	.81	.56
3-way	.89	.84	.63

Note: Krippendorff's α (nominal) on verbatim n-shot prompts. Each GPT generation treated as a rater.

Table 1: Cross-generation agreement (Krippendorff's α).

Adjacent generations agree well: $\alpha = 0.91$ (4o–4.1) and 0.89 (4.1–5.4) on P, 0.88 and 0.84 on L_{I}. Agreement decays with generational distance — 4o–5.4 is consistently the lowest pair — and with task difficulty: L_{II} reaches only $\alpha = 0.56$–0.71. Since accuracy improves monotonically across generations, the disagreement reflects improvement rather than drift: a newer model from the same family can be expected to produce results at least as reliable as substituting one trained human annotator for another.
