# Supplementary material for: Much Ado about Prompting: LLM classification of text messages from experiments
Source: PLoS One. 2026 Aug 4;21(8):e0354757. doi: 10.1371/journal.pone.0354757 (PMC13436748; doi:10.1371/journal.pone.0354757)
Supplement: Supplementary file 8 [file pone.0354757.s008.pdf]

S8 Appendix — API and Implementation Details

Supporting Information for "Much Ado about Prompting: LLM Classification of Text Messages from Experiments"

Can Çelebi   Stefan P. Penczynski

1 API and Implementation Details

All classifications are performed via API calls to four providers. Table **??** summarises the configuration per pool.

Pool	Provider	Models	Endpoints
Azure OpenAI	Microsoft	GPT 5.4 family	7
Together AI	Together	Qwen 3.5 9B	1
OpenRouter	Alibaba	Qwen 3.5 397B/122B/35B/27B	1

Table 1: API pools.

Temperature and determinism. Temperature is set to 0 for all non-reasoning runs, maximising output determinism. Reasoning runs require temperature 1 (a constraint imposed by GPT models; applied uniformly across all providers for consistency). A fixed seed (31) is set for all calls where the API supports it.

Structured output. All calls enforce structured JSON output via a task-specific JSON schema passed in the `response_format` parameter. This ensures that every response is machine-parseable without post-processing.

Logprobs. Token-level log-probabilities are collected for all non-reasoning runs (`top_logprobs` = 5 for GPT, Together, and OpenRouter; 10 for Foundry). Logprobs are disabled for reasoning runs, as GPT does not support them when reasoning is active.

Batching and rate limiting. Requests are issued in parallel batches of 35 (Azure pools, across multiple endpoints) or 10 (Together AI, OpenRouter, single endpoint), with a 1-second inter-batch delay. Token usage is tracked per endpoint to stay within rate limits (250,000 tokens/minute per Azure resource); the runner rotates to the least-loaded endpoint within each pool.

Error handling and retries. Transient errors (HTTP 5xx, connection resets) trigger rotation across endpoints without delay; if all endpoints fail, exponential backoff begins at 2 seconds. Content-filter rejections (HTTP 400) are retried once on each available endpoint; on OpenRouter, items rejected by Alibaba's filter are automatically retried on an alternative provider (AtlasCloud) without logprobs. After 20 consecutive batch failures, execution pauses for 10 minutes before resuming. Maximum retries per item: 5 or the pool size, whichever is larger.

Output format. Each classified item is written as a single JSON line to a task-specific JSONL file, recording: the item ID, dataset, prompt variant, model output,

timestamp, token counts, logprobs (when available), and an error flag. Files are append-only and resumable — restarting the runner skips items already present in the output file.
