# Supplementary material for: Much Ado about Prompting: LLM classification of text messages from experiments
Source: PLoS One. 2026 Aug 4;21(8):e0354757. doi: 10.1371/journal.pone.0354757 (PMC13436748; doi:10.1371/journal.pone.0354757)
Supplement: Supplementary file 9 [file pone.0354757.s009.pdf]

S9 Appendix — Cost and Latency

Supporting Information for "Much Ado about Prompting: LLM Classification of Text Messages from Experiments"

Can Çelebi   Stefan P. Penczynski

1 Cost and Latency

Table **??** compares the cost of reasoning and examples for GPT 5.4 at Azure pricing ($2.25/M input, $13.00/M output). Reasoning tokens are charged at the output rate (5.78× more expensive than input tokens), while examples add only input-rate tokens. The "Ratio" column expresses each intervention's cost as a fraction of the average 0-shot prompt cost ($3.83 per 1,000 calls, averaging over P=548, L_{I}=2,214, L_{II}=2,346 input tokens).

	μ_{tokens}	Cost	Ratio
Reasoning			
Low	113	1.47	0.38×
Medium	322	4.19	1.09×
High	746	9.70	2.53×
Extra-high	2,479	32.23	8.42×
Examples			
P examples	746	1.68	0.44×
L_{I} examples	599	1.35	0.35×
L_{II} examples	521	1.17	0.31×

Note: GPT 5.4 Azure pricing as of April 2026: $2.25/M input tokens, $13.00/M output tokens. Cost in USD per 1,000 calls. Reasoning tokens are charged at the output rate (5.78× the input rate); examples are charged at the input rate. Ratio = intervention cost / average 0-shot prompt cost (3.83 per 1,000 calls). `Low` reasoning and adding examples cost a similar fraction of the prompt (≈0.3–0.4×), but examples deliver far larger accuracy gains. `High` reasoning costs 2.5× the entire prompt; `extra-high` costs 8.4×.

Table 1: Cost comparison: reasoning vs. examples (GPT 5.4).

Table **??** reports the latency cost of reasoning. For GPT 5.4, `low` reasoning doubles median response time (1.4 s → 3.5 s); `high` is 8× slower (11.6 s); `extra-high` is 21× slower (30.2 s). Qwen reasoning is far more expensive in time: 50–58× slower (median), reflecting less optimised reasoning infrastructure. Adding examples, by contrast, adds zero extra latency — input tokens are processed in parallel.

	Reasoning	Median	Slowdown
GPT 5.4	off	1.4	1.0×
	low	3.5	2.5×
	medium	6.8	4.7×
	high	11.6	8.1×
	extra-high	30.2	21.0×
GPT 5.4-nano	off	1.0	1.0×
	low	1.3	1.3×
	high	1.9	1.9×
	extra-high	5.4	5.5×
Qwen 397B	off	0.8	1.0×
	on	49.0	58.6×
Qwen 9B	off	0.6	1.0×
	on	38.3	69.5×

Note: Median end-to-end response time per API call in seconds. Slowdown relative to no-reasoning baseline for each model. At 2,101 items, `extra-high` reasoning on GPT 5.4 takes ≈17.5 hours vs. ≈50 minutes without reasoning.

Table 2: Reasoning latency by model and effort level.

Adding examples costs roughly the same fraction of the prompt as `low` reasoning (both ≈37–38%) but delivers far larger accuracy gains — for example, L_{II} minimal accuracy rises from 19% to 60% with examples (Section 6.3), whereas reasoning at any level produces at most single-digit improvements on the same task (Section 6.4). The practical recommendation is to invest in prompt content (examples, detailed instructions) before enabling reasoning.
