# Supplementary material for: Much Ado about Prompting: LLM classification of text messages from experiments
Source: PLoS One. 2026 Aug 4;21(8):e0354757. doi: 10.1371/journal.pone.0354757 (PMC13436748; doi:10.1371/journal.pone.0354757)
Supplement: Supplementary file 10 [file pone.0354757.s010.pdf]

S10 Appendix — Codebook-to-Prompt Pipeline: Prompts

Supporting Information for "Much Ado about Prompting: LLM Classification of Text Messages from Experiments"

Can Çelebi Stefan P. Penczynski

1 Codebook-to-Prompt Pipeline: Prompts

All LLM-assisted pipeline transformations in this appendix — filtering, compression, and the verbose and noise expansions — were produced with Claude Opus 4.6 at temperature 0 via Azure AI Foundry, applied once per codebook section. The compression and filtering outputs were validated by checking that a list of task-specific key terms survived the transformation, regenerating otherwise.

1.1 Filtering Prompt

The following prompt was used with Claude Opus 4.6 to filter each raw codebook, removing procedural content while preserving all classification-relevant material.

```
# General Task
- Filter a human classification codebook to retain only content
relevant for an LLM classification prompt.

# Instructions
- Read the codebook below and remove all content that is only
relevant to human annotators working in a laboratory setting.
- REMOVE the following types of content:
  + Greetings, welcomes, and introductory pleasantries
  + Time estimates (``the experiment will take about 20
minutes'')
  + Payment, remuneration, bonus, and reward details
  + Excel sheet, spreadsheet, or file handling instructions
  + Column descriptions, data entry instructions, formatting
guidance
  + Instructions to work independently or not discuss with others
  + Instructions to review or double-check coding
  + References to instruction sheets, quizzes, or other documents
  + Meta-instructions (``the next page describes...'', ``we now
describe...'')
  + Timeout warnings and deadline-related instructions
  + Separator lines (dashes, horizontal rules)
  + ``Let's illustrate...''  or similar transitional phrases
before examples
- KEEP the following types of content:
  + Experiment mechanics (what players do, payoffs, choices,
communication)
  + Classification definitions (what each label means, conditions
for each category)
  + Examples with their classifications and explanatory remarks
  + Any domain-specific concepts needed for classification
- Light reframing:  where the original text addresses subjects
in second person (``You will...''), reframe to third person
(``Participants will...'').
- Do NOT add any information not present in the original.
- Do NOT summarise or shorten the kept content.
- Preserve the section structure with clear headers.
```

Figure 1: Filtering prompt used in Step 1 of the pipeline.

1.2 Compression Prompt

The following prompt was applied iteratively to generate compressed variants. Each application halves the text: V $\rightarrow$ C1 ($\approx$50%) and C1 $\rightarrow$ C2 ($\approx$25%). A list of task-specific key terms was enforced to prevent loss of essential vocabulary during compression.

```
# General Task
- Create an expert-level summary of the following experiment
description.  This text has already been filtered to contain only
classification-relevant content.

# Instructions
- Shorten the text below to approximately half of its current
length.
- ONLY REMOVE information.  Do NOT add any information,
inferences, or clarifications not explicitly stated in the
original text.
- You MUST keep the following terms exactly as they appear:
{preserved_terms}
- Prioritize keeping (in this order):
  + What participants choose and what happens as a result (game
mechanics, payoffs)
  + How participants communicate (message protocol)
  + The number and roles of participants
  + The objective of the game
- When removing content, prefer dropping:
  + Derived or illustrative quantities
  + Elaborations that restate a mechanic already captured
  + Treatment-specific details (e.g., number of rounds, group
re-matching)
- Do not paraphrase unless necessary for coherence after removing
a sentence.
- Present the output as a list of statements.  Each statement
should be at most 2 sentences long.  Start each statement on a
new line with ``- ''.
```

Figure 2: Compression prompt used in Step 3 of the pipeline (experiment context version).

A parallel prompt was used for theory sections, prioritizing level/type definitions and inter-level reasoning relationships over formal notation and numerical parameters.

1.3 Verbose and Noise Prompts

To test whether models respond to informational content or merely to prompt length (design in Appendix 1.5; results in Appendix 1), each verbatim section was rewritten in two ways with Claude Opus 4.6. The *verbose* variant (V_{ex}) elaborates the existing content without adding new information (word count +50–275%); the length-matched *noise* variant (V_{noise}) instead pads the same section to the verbose length with topic-irrelevant filler (red-squirrel ecology) interspersed at the sentence level. The contrast isolates the effect of relevance from that of length. The two generation prompts are shown in Figures **??** and **??**; both were applied per section (experiment context, theory, classification instructions), and all original wording, structure, and category definitions were preserved.

# General Task
- Expand the following classification prompt section, making its existing content fuller and more explicit without adding any new information.

# Instructions
- Increase the length of the text below by roughly 50--275%, keeping its meaning unchanged.
- ONLY ELABORATE on information already present. Do NOT introduce new rules, categories, criteria, or conceptual content not stated in the original.
- Permitted expansions:
  + Provide more detail about information already stated
  + Restate key points in alternative phrasings
  + Make implicit criteria explicit and spell out boundary cases already implied
- Do NOT change any classification definition, label, or decision rule.
- Preserve the section structure, headers, and the order of the level/category descriptions.
- Keep all task-specific key terms exactly as they appear: {preserved_terms}

# Text to expand
{text}

Figure 3: Verbose-expansion prompt used with Claude Opus 4.6 to generate the V_{ex} variants.

# General Task
- Lengthen the following classification prompt by inserting topic-irrelevant filler, without changing any of its original content.

# Instructions
- Insert sentences of irrelevant factual content (ecology of the red squirrel, *Sciurus vulgaris*) into the text below.
- Add filler until the total word count matches the verbose (V_{ex}) variant of the same section.
- Intersperse the filler at the sentence level --- distribute it between existing sentences and bullet points rather than appending it in one block.
- Do NOT modify, remove, paraphrase, or reorder any of the original text. Every original sentence, header, label, and classification rule must remain exactly as written.
- The inserted content must be entirely unrelated to the classification task (no strategic reasoning, games, voting, or promises).

# Text to pad
{text}

Figure 4: Noise-injection prompt used with Claude Opus 4.6 to generate the length-matched V_{noise} control variants.
