# Supplementary material for: Much Ado about Prompting: LLM classification of text messages from experiments
Source: PLoS One. 2026 Aug 4;21(8):e0354757. doi: 10.1371/journal.pone.0354757 (PMC13436748; doi:10.1371/journal.pone.0354757)
Supplement: Supplementary file 11 [file pone.0354757.s011.pdf]

S11 Appendix — Prompt Template Rationale

Supporting Information for “Much Ado about Prompting: LLM Classification of Text Messages from Experiments”

Can Çelebi Stefan P. Penczynski

1 Prompt Template Rationale

Our prompt template (Figure 1) was designed following established guidelines for effective prompt design [1, 2, 3, 4]. Research has shown that using instructions written for human annotators directly as prompts leads to poor LLM performance [5], whereas reframing them into structured templates substantially improves performance [4]. Each component of the template is motivated by specific findings in the prompting literature:

- **General Task.** A high-level task specification using key terms (“classify”, “message”, “promise”) primes the model toward the intended output without specifying how to accomplish the task [6, 7, 3].
- **Role Persona.** Assigning a domain-specific persona (“behavioural economist specialised in level-k reasoning”) acts as an augmented task specification, drawing on the model’s pre-trained knowledge of expert reasoning patterns [6, 8]. Persona assignment has been shown to elevate performance comparably to chain-of-thought prompting, though it may surface biases from training data [9].
- **Context.** Background information about the experiment and, for L-tasks, the underlying theoretical framework. Previous studies document positive effects from including background details [10, 11, 12, 13]. In our pipeline, the verbatim codebook text is retained as the baseline and then systematically compressed to test how much detail is needed (Section 5.1).
- **Classification Task.** Expert-crafted category definitions from the original codebook, restructured into itemised lists. Expert label descriptions significantly outperform both no descriptions and automatically generated ones [14], and expert task descriptions surpass basic NLP-style instructions [7, 15, 16].
- **Examples.** Worked examples from the original codebook, included verbatim where available. Included as a dedicated section to allow independent variation in the factorial design.
- **Constraints and Output Format.** Enforce structured JSON output for consistent, machine-parseable results [6, 4, 3]. **(author?)** [17] find that format restrictions can degrade reasoning performance but actually improve accuracy on classification tasks, supporting the use of constrained JSON output in our setting.

Segmenting instructions into itemised lists improves model comprehension [4]. We use Markdown formatting throughout, as it supports itemisation and subtitles with minimal token overhead. **(author?)** [18] compare plain text, Markdown, JSON, and YAML formats across GPT-3.5 and GPT-4, finding that GPT-3.5 performance varies by up to 40% across formats while GPT-4 is substantially more robust — consistent with our finding that format sensitivity diminishes with model scale.

References

[1]Zhao WX, Zhou K, Li J, Tang T, Wang X, Hou Y, et al. A survey of large language models. arXiv preprint arXiv:230318223. 2023.

[2]Ziems C, Held W, Shaikh O, Chen J, Zhang Z, Yang D. Can large language models transform computational social science? Computational Linguistics. 2024:1-55.

[3]White J, Fu Q, Hays S, Sandborn M, Olea C, Gilbert H, et al. A prompt pattern catalog to enhance prompt engineering with chatgpt. arXiv preprint arXiv:230211382. 2023.

[4]Mishra S, Khashabi D, Baral C, Choi Y, Hajishirzi H. Reframing Instructional Prompts to GPTk's Language. arXiv preprint arXiv:210907830. 2021.

[5]Efrat A, Levy O. The turking test: Can language models understand instructions? arXiv preprint arXiv:201011982. 2020.

[6]Reynolds L, McDonell K. Prompt programming for large language models: Beyond the few-shot paradigm. In: Extended Abstracts of the 2021 CHI Conference on Human Factors in Computing Systems; 2021. p. 1-7.

[7]Mishra S, Khashabi D, Baral C, Hajishirzi H. Cross-task generalization via natural language crowdsourcing instructions. arXiv preprint arXiv:210408773. 2021.

[8]Kong A, Zhao S, Chen H, Li Q, Qin Y, Sun R, et al. Better zero-shot reasoning with role-play prompting. arXiv preprint arXiv:230807702. 2023.

[9]Salewski L, Alaniz S, Rio-Torto I, Schulz E, Akata Z. In-Context Impersonation Reveals Large Language Models' Strengths and Biases. Advances in Neural Information Processing Systems. 2024;36.

[10]Chae Y, Davidson T. Large language models for text classification: From zero-shot learning to fine-tuning. Sociological Methods & Research. 2025. doi:10.1177/00491241251325243.

[11]Savelka J, Ashley KD, Gray MA, Westermann H, Xu H. Can gpt-4 support analysis of textual data in tasks requiring highly specialized domain expertise? arXiv preprint arXiv:230613906. 2023.

[12]Yuan J, Bao P, Chen Z, Yuan M, Zhao J, Pan J, et al. Advanced prompting as a catalyst: Empowering large language models in the management of gastrointestinal cancers. The Innovation. 2023.

[13]Clavié B, Ciceu A, Naylor F, Soulié G, Brightwell T. Large language models in the workplace: A case study on prompt engineering for job type classification. In: International Conference on Applications of Natural Language to Information Systems. Springer; 2023. p. 3-17.

[14]Peskine Y, Korenčić D, Grubisic I, Papotti P, Troncy R, Rosso P. Definitions

Matter: Guiding GPT for Multi-label Classification. In: Findings of the Association for Computational Linguistics: EMNLP 2023; 2023. p. 4054-63.
[15]Logan IV RL, Balažević I, Wallace E, Petroni F, Singh S, Riedel S. Cutting down on prompts and parameters: Simple few-shot learning with language models. arXiv preprint arXiv:210613353. 2021.
[16]Schick T, Schütze H. It's not just size that matters: Small language models are also few-shot learners. arXiv preprint arXiv:200907118. 2020.
[17]Tam ZR, Wu CK, Tsai YL, Lin CY, Lee Hy, Chen YN. Let Me Speak Freely? A Study on the Impact of Format Restrictions on Performance of Large Language Models. In: Proceedings of the 2024 Conference on Empirical Methods in Natural Language Processing: Industry Track; 2024. .
[18]He J, Rungta M, Koleczek D, Sekhon A, Hasan SS. Does Prompt Formatting Have Any Impact on LLM Performance? arXiv preprint arXiv:241110541. 2024.
