# Supplementary material for: Much Ado about Prompting: LLM classification of text messages from experiments
Source: PLoS One. 2026 Aug 4;21(8):e0354757. doi: 10.1371/journal.pone.0354757 (PMC13436748; doi:10.1371/journal.pone.0354757)
Supplement: Supplementary file 12 [file pone.0354757.s012.pdf]

S12 Appendix — Prior Work Comparison

Supporting Information for "Much Ado about Prompting: LLM Classification of Text Messages from Experiments"

Can Çelebi Stefan P. Penczynski

1 Prior Work Comparison

Table 1 compares our study with the most closely related prior work on prompt design for LLM text classification. Past work on prompt design has explored subsets of the space we cover—specifically content detail, surface variation, and model scale (see Table 1). When placed onto this three-dimensional map, their isolated findings align perfectly with our unified results.

Studies varying content detail [1, 2, 3] recover the same patterns we report: different types of information can substitute for one another, and excessive detail overloads smaller models. Conversely, studies exploring surface variation — such as formatting, framing, and phrasing — [4, 5] find that these choices are largely inert when content is well-specified, but cause erratic behavior in smaller models. **(author?)** [6] varies both, noting that detailed definitions aid ambiguous tasks while surface variations shift the output distribution.

None of these studies, however, cross all three axes. Crucially, none test how surface variation and prompt detail interact at frontier model scale—which is the exact gap our design fills. We take each study in turn.

Study	Dimensions varied	Models	Scale	Tasks (labels)	Content[a]	Prompt[b]	Key finding
**(author?)** [1]	Description depth (3) × examples (0/15)	5	13B–GPT-4	Fatherhood roles (6)	Yes	∼40–1,200	Examples dominant; descriptions partially substitute but add less when examples present
**(author?)** [2]	6 per-label fields[c] (sequential removal)	4	7–12B	Political events (8–142)	Partial	∼600–3,900	Small models rely on label names over definitions; zero-shot only
**(author?)** [3]	Codebook none/short/full × examples (0/1/2)	3	7–27B	Gun violence frames (9–13)	Yes	∼130–1,150	Codebook helps larger model; examples on top of codebook neutral or hurt
**(author?)** [4]	Persona, context, coding strategy, reasoning, justification (864 combos)[d]	2	7–12B	Climate coding (2)	Partial	∼100–200	Coding strategy dominant; persona and context not significant
**(author?)** [6]	Definition × output format × explanation × length (2^4)	4	7B–GPT-4o	Toxicity, sentiment, stance, frames (2–9)	Yes	∼25–325	Definitions help on ambiguous tasks; explanation shifts label distributions
**(author?)** [5]	Persona × reasoning × examples (0/n)	6	∼20–72B	Approval, sentiment, distance, topics (2–8)	No	∼80–900	No single approach uniformly best; persona and reasoning erratic across models
**This study**	**Content × format × framing × examples × reasoning**	**4**	**9B–GPT 5.4**	**Promise, level-k reasoning (2–6)**	**Yes**	**∼110–1,500**	**Content dominant; surface irrelevant on rich prompts; scale moderates all dimensions**

[a]Content richness varied independently of other dimensions. "Partial" = content varied but compensation effects not testable. [b]Approximate word count of the prompt (excluding the item text), from the smallest to the richest variant tested. [c]Label name, definition, clarification, negative clarification, positive example, negative example. [d]8 dimensions with 2–3 levels each: persona, context, task specification, coding strategy, coding elements, reasoning trigger, reasoning steps, justification.

Table 1: Comparison with prior work on prompt design for LLM text classification.

(author?) [1] provide the clearest prior evidence for information substitutability: adding category descriptions helps most when examples are absent, and vice versa. We extend this by decomposing content into four functional components (experiment context, theory, classification instructions, examples) at multiple compression levels rather than a binary presence/absence, by crossing content with formatting, framing, and reasoning, and by testing across a wider model range including frontier models. Their prompts range from ∼40 to ∼1,200 words, comparable to ours when examples are included, while focusing on two dimensions (description depth and shot count).

(author?) [2] ablate six per-label codebook fields and find that small models fall back on label name heuristics when definitions are removed. This is consistent with our finding that smaller models are more dependent on detailed classification instructions. We extend their work by testing larger models (up to GPT 5.4), where this dependence diminishes, and by varying content richness independently of other prompt dimensions rather than in sequential removal. Their prompts reach ∼3,900 words for the 142-label manifesto task, the largest in this literature; their study focuses on two smaller models (7–12B), leaving open the question of whether frontier models would show the same sensitivity.

(author?) [3] find that providing the full codebook helps the largest model in their study (Gemma 27B) but that adding examples on top of the codebook is neutral or harmful. This parallels our finding that examples compensate for missing content but add little when the codebook is already rich. The scale interaction they observe — the larger model benefits from detail while the smaller ones are confused by it — matches ours in direction, though it is confined to small-to-mid open models (7–27B) and does not reach frontier scale. We complement their coarse codebook contrast (none/short/full) by testing finer graded compression levels, and extend the model range up to GPT 5.4.

(author?) [4] systematically vary eight prompt engineering dimensions and find that the coding strategy (i.e., how detailed the classification instructions are) is the dominant factor — consistent with our finding that classification instructions account for the majority of explained variance. Their finding that persona and context are not significant mirrors our framing results on well-specified prompts. We extend by independently varying content richness, which their design holds approximately constant within a compact ∼100–200 word prompt range, and by testing whether their conclusions hold at larger model scales.

(author?) [6] use a full 2^4 factorial on four prompt features and find that category definitions help on ambiguous multi-class tasks but not on clear binary ones. This parallels our distinction between learning-heavy tasks (where detailed instructions are essential) and recognition-heavy tasks (where model choice matters more than content). They explicitly call for future work testing interactions between prompt dimensions; our factorial design with interaction analysis addresses this directly. Their prompts span ∼25–325 words, placing them near the lower end of the specification range where our results would predict sensitivity to surface variations to be highest.

(author?) [5] hold codebook content fixed — and relatively rich, with label definitions, decision rules, and worked examples — and vary only model choice, persona, reasoning, and examples across six open-weight models (∼20–72B, quantised). Their

headline finding — that interaction effects dominate main effects and no single approach is uniformly best — shows that surface variations can remain erratic even on richer content when the models are small open-weight ones, consistent with our finding that smaller models retain a surface sensitivity that frontier models do not. We extend by varying content richness directly and by testing frontier-scale proprietary models, where this inconsistency largely disappears — a comparison their fixed-content, open-weight design cannot make. Their prompt range (∼80–900 words) is moderate, and their focus on open-weight models at quantised precision leaves open the question of how the same patterns extend to frontier-scale proprietary models.

References

[1]Lupo L, Magnusson M, Hovy D, Naurin E, Wangnerud L. Towards Human-Level Text Coding with LLMs: The Case of Fatherhood Roles in Public Policy Documents. arXiv preprint arXiv:231111844. 2024.

[2]Halterman A, Keith KA. Codebook LLMs: Evaluating LLMs as Measurement Tools for Political Science Concepts. Political Analysis. 2025.

[3]Ruckdeschel M. Just Read the Codebook! Make Use of Quality Codebooks in Zero-Shot Classification of Multilabel Frame Datasets. In: Proceedings of the 31st International Conference on Computational Linguistics (COLING); 2025. p. 6317-37.

[4]Reich A, Thoms C, Schrimpf T. Introducing HALC: A General Pipeline for Finding Optimal Prompting Strategies for Automated Coding with LLMs in the Computational Social Sciences. arXiv preprint arXiv:250721831. 2025.

[5]McLaren L, Cross JP, Krakowska Z, Rauner R, Schoonvelde M. Magic Words or Methodical Work? Challenging Conventional Wisdom in LLM-Based Political Text Annotation. arXiv preprint arXiv:260326898. 2026.

[6]Atreja S, Ashkinaze J, Li L, Mendelsohn J, Hemphill L. Prompt Design Matters for Computational Social Science Tasks but in Unpredictable Ways. In: Proceedings of the International AAAI Conference on Web and Social Media. vol. 19; 2025. p. 122-45.
