# Supplementary material for: Much Ado about Prompting: LLM classification of text messages from experiments
Source: PLoS One. 2026 Aug 4;21(8):e0354757. doi: 10.1371/journal.pone.0354757 (PMC13436748; doi:10.1371/journal.pone.0354757)
Supplement: Supplementary file 13 [file pone.0354757.s013.pdf]

S13 Appendix — F1 Metric Replication

Supporting Information for "Much Ado about Prompting: LLM Classification of Text Messages from Experiments"

Can Çelebi Stefan P. Penczynski

1 F1 Metric Replication

The main text reports accuracy throughout. This appendix replicates the headline tables and information surfaces using F1, to verify that the qualitative findings do not depend on the choice of scoring metric.

Metric. We report support-weighted macro F1: per-class F1 averaged with weights equal to each class's support (the number of items where that class is a valid answer). Micro F1 equals accuracy by definition for single-label tasks and is uninformative here; unweighted macro F1 over-penalises rare levels, which is inappropriate because strategic-reasoning level distributions are systematically skewed toward low levels [1, 2] and levels 4–5 appear in only a handful of L_{II} items. Weighted F1 mirrors accuracy's implicit frequency weighting while still decomposing per-class precision and recall. For L_{II} we preserve the interval-membership agreement rule used throughout the main text: TP(c) = prediction = c with c in the interval; FP(c) = prediction = c with c outside the interval; FN(c) is counted only when the prediction itself falls outside the interval, so picking one valid level over another does not generate an FN.

Confidence intervals. Per-cell F1 values carry 95% item-level bootstrap intervals ($B = 1{,}000$, seed 42). F1 deltas carry paired bootstrap intervals: a single resample of item indices is applied to both conditions before the delta is computed, yielding tighter within-model intervals.

1.1 F1 at minimal vs verbatim content

	Minimal			Verbatim		
	P	L_{I}	L_{II}	P	L_{I}	L_{II}
GPT 5.4	.73 [.71, .75]	.47 [.42, .51]	.51 [.48, .55]	.74 [.71, .76]	.81 [.77, .84]	.78 [.75, .81]
GPT 5.4-nano	.60 [.57, .62]	.50 [.46, .54]	.53 [.50, .56]	.64 [.61, .66]	.68 [.64, .72]	.65 [.62, .69]
Qwen 397B	.68 [.66, .71]	.50 [.46, .54]	.42 [.38, .45]	.62 [.60, .65]	.82 [.79, .86]	.73 [.70, .76]
Qwen 9B	.62 [.60, .65]	.53 [.49, .57]	.40 [.37, .43]	.62 [.59, .65]	.71 [.67, .75]	.56 [.52, .61]

Note: Support-weighted macro F1 for all three tasks with 95% item-level bootstrap CI ($B = 1{,}000$) in brackets. L_{II} uses the interval-membership agreement rule from the main text. Compare to Table 3.

Table 1: F1 at the minimal and verbatim prompts.

1.2 F1 with the full codebook

	P	L_{I}	L_{II}
GPT 5.4	.88 [.86, .90]	.84 [.80, .87]	.78 [.75, .81]
GPT 5.4-nano	.72 [.69, .74]	.77 [.73, .81]	.65 [.61, .69]
Qwen 397B	.87 [.85, .89]	.83 [.80, .86]	.75 [.72, .78]
Qwen 9B	.78 [.77, .80]	.82 [.79, .86]	.61 [.57, .65]

Note: Support-weighted macro F1 on verbatim content + n-shot examples, no reasoning, with 95% item-level bootstrap CI. Compare to Table 6.

Table 2: F1 with the full codebook.

1.3 F1 deltas: examples and content

Tables 3–6 replicate the examples-vs-content decomposition (main-text Table 5 and Appendix 1.2) under weighted F1 with paired bootstrap CIs.

	P	L_{I}	L_{II}
Δ_{Ex}	+.17 [+.14, +.19]	+.38 [+.33, +.44]	+.17 [+.14, +.22]
$\Delta_{\text{Ex}} - \Delta_{\text{Content}}$	+.16 [+.14, +.18]	+.04 [+.01, +.08]	-.09 [-.13, -.05]
$\Delta_{\text{Ex}\mid\text{Content}}$	+.14 [+.12, +.17]	+.03 [+.01, +.06]	-.00 [-.02, +.02]
$\Delta_{\text{Content}\mid\text{Ex}}$	-.02 [-.03, -.01]	-.01 [-.04, +.01]	+.09 [+.05, +.13]

Note: F1 deltas with 95% paired bootstrap CI (B = 1,000). A CI excluding zero indicates a directionally reliable effect. Compare to Table 5.

Table 3: GPT 5.4: effect of examples (F1 metric).

	P	L_{I}	L_{II}
Δ_{Ex}	+.09 [+.06, +.12]	+.22 [+.17, +.27]	+.05 [+.01, +.09]
$\Delta_{\text{Ex}} - \Delta_{\text{Content}}$	+.05 [+.03, +.08]	+.04 [-.00, +.09]	-.07 [-.11, -.03]
$\Delta_{\text{Ex}\mid\text{Content}}$	+.08 [+.05, +.10]	+.09 [+.06, +.13]	-.00 [-.03, +.02]
$\Delta_{\text{Content}\mid\text{Ex}}$	+.03 [+.01, +.05]	+.05 [+.01, +.09]	+.07 [+.03, +.11]

Table 4: GPT 5.4-nano: effect of examples (F1 metric).

	P	L_{I}	L_{II}
Δ_{Ex}	+.19 [+.16, +.21]	+.33 [+.27, +.37]	+.26 [+.22, +.30]
$\Delta_{\text{Ex}} - \Delta_{\text{Content}}$	+.24 [+.21, +.27]	+.00 [-.03, +.03]	-.05 [-.09, -.02]
$\Delta_{\text{Ex}\mid\text{Content}}$	+.24 [+.21, +.27]	+.01 [-.02, +.03]	+.02 [-.00, +.04]
$\Delta_{\text{Content}\mid\text{Ex}}$	-.00 [-.01, +.01]	+.01 [-.02, +.04]	+.08 [+.04, +.10]

Table 5: Qwen 397B: effect of examples (F1 metric).

	P	L_{I}	L_{II}
Δ_{Ex}	+.16 [+.13, +.19]	+.13 [+.07, +.18]	+.26 [+.22, +.31]
$\Delta_{\text{Ex}} - \Delta_{\text{Content}}$	+.16 [+.14, +.19]	-.05 [-.10, -.01]	+.09 [+.06, +.13]
$\Delta_{\text{Ex}\mid\text{Content}}$	+.16 [+.14, +.19]	+.12 [+.08, +.15]	+.05 [+.02, +.08]
$\Delta_{\text{Content}\mid\text{Ex}}$	+.00 [-.01, +.02]	+.17 [+.13, +.21]	-.05 [-.08, -.01]

Table 6: Qwen 9B: effect of examples (F1 metric).

1.4 F1 deltas: content vs reasoning

Tables 7–10 replicate the content-vs-reasoning decomposition (main-text Table 7 and Appendix 1.3) under weighted F1 with paired bootstrap CIs.

	P	L_{I}	L_{II}
Δ_{Rea}	+.09 [+.07, +.12]	-.16 [-.21, -.11]	+.17 [+.13, +.21]
$\Delta_{\text{Rea}} - \Delta_{\text{Content}}$	+.09 [+.07, +.11]	-.50 [-.55, -.44]	-.10 [-.14, -.06]
$\Delta_{\text{Content}\mid\text{Rea}}$	-.12 [-.14, -.09]	+.51 [+.45, +.57]	+.10 [+.06, +.14]
$\Delta_{\text{Rea}\mid\text{Content}}$	-.03 [-.05, -.01]	+.02 [-.01, +.04]	+.00 [-.03, +.03]

Note: F1 deltas with 95% paired bootstrap CI. GPT uses high reasoning effort; Qwen uses `reasoning: on`. Compare to Table 7.

Table 7: GPT 5.4: content vs. reasoning substitutability (F1 metric).

	P	L_{I}	L_{II}
Δ_{Rea}	+.10 [+.07, +.12]	+.13 [+.08, +.18]	-.09 [-.13, -.06]
$\Delta_{\text{Rea}} - \Delta_{\text{Content}}$	+.05 [+.03, +.08]	-.05 [-.10, +.00]	-.22 [-.26, -.17]
$\Delta_{\text{Content}\mid\text{Rea}}$	-.00 [-.02, +.02]	+.12 [+.07, +.17]	+.21 [+.15, +.26]
$\Delta_{\text{Rea}\mid\text{Content}}$	+.05 [+.03, +.07]	+.07 [+.03, +.12]	-.01 [-.04, +.03]

Table 8: GPT 5.4-nano: content vs. reasoning substitutability (F1 metric).

	P	L_{I}	L_{II}
Δ_{Rea}	+.00 [-.02, +.02]	+.05 [+.01, +.10]	-.02 [-.05, +.01]
$\Delta_{\text{Rea}} - \Delta_{\text{Content}}$	+.06 [+.04, +.08]	-.28 [-.33, -.23]	-.33 [-.37, -.29]
$\Delta_{\text{Content}\mid\text{Rea}}$	+.05 [+.03, +.07]	+.28 [+.24, +.33]	+.33 [+.28, +.37]
$\Delta_{\text{Rea}\mid\text{Content}}$	+.10 [+.08, +.13]	+.01 [-.02, +.03]	-.00 [-.04, +.03]

Table 9: Qwen 397B: content vs. reasoning substitutability (F1 metric).

	P	L_{I}	L_{II}
Δ_{Rea}	+.04 [+.02, +.06]	+.07 [+.01, +.11]	-.11 [-.15, -.08]
$\Delta_{\text{Rea}} - \Delta_{\text{Content}}$	+.04 [+.02, +.06]	-.12 [-.17, -.06]	-.28 [-.33, -.24]
$\Delta_{\text{Content}\mid\text{Rea}}$	+.06 [+.04, +.09]	+.21 [+.16, +.26]	+.35 [+.31, +.40]
$\Delta_{\text{Rea}\mid\text{Content}}$	+.10 [+.08, +.13]	+.10 [+.05, +.14]	+.07 [+.03, +.11]

Table 10: Qwen 9B: content vs. reasoning substitutability (F1 metric).

1.5 F1 deltas: reasoning effect on verbatim content (all models)

	0-shot			n-shot		
	P	L_{I}	L_{II}	P	L_{I}	L_{II}
GPT 5.4	-.03 [-.05, -.01]	+.02 [-.01, +.04]	+.00 [-.03, +.03]	+.01 [+.00, +.03]	+.02 [-.00, +.04]	+.03 [-.00, +.06]
GPT 5.4-nano	+.05 [+.02, +.08]	+.07 [+.03, +.12]	-.01 [-.04, +.03]	+.11 [+.09, +.13]	+.02 [-.02, +.05]	-.02 [-.05, +.01]
Qwen 397B	+.10 [+.08, +.13]	+.01 [-.01, +.03]	-.00 [-.03, +.03]	+.03 [+.02, +.05]	+.03 [+.01, +.05]	+.02 [+.00, +.05]
Qwen 9B	+.10 [+.08, +.13]	+.10 [+.06, +.14]	+.07 [+.03, +.11]	+.09 [+.07, +.11]	+.02 [-.01, +.04]	+.07 [+.03, +.11]

Note: F1 deltas (verbatim + reasoning minus verbatim) with 95% paired bootstrap CI. GPT models use high reasoning effort; Qwen models use `reasoning: on`. Compare to Table 2.

Table 11: Effect of reasoning on verbatim content (F1 metric).

1.6 F1 information surfaces

Figures 1 and 2 replicate the L_{I} and L_{II} information surfaces (Section 6.1) under F1 scoring for all four models.

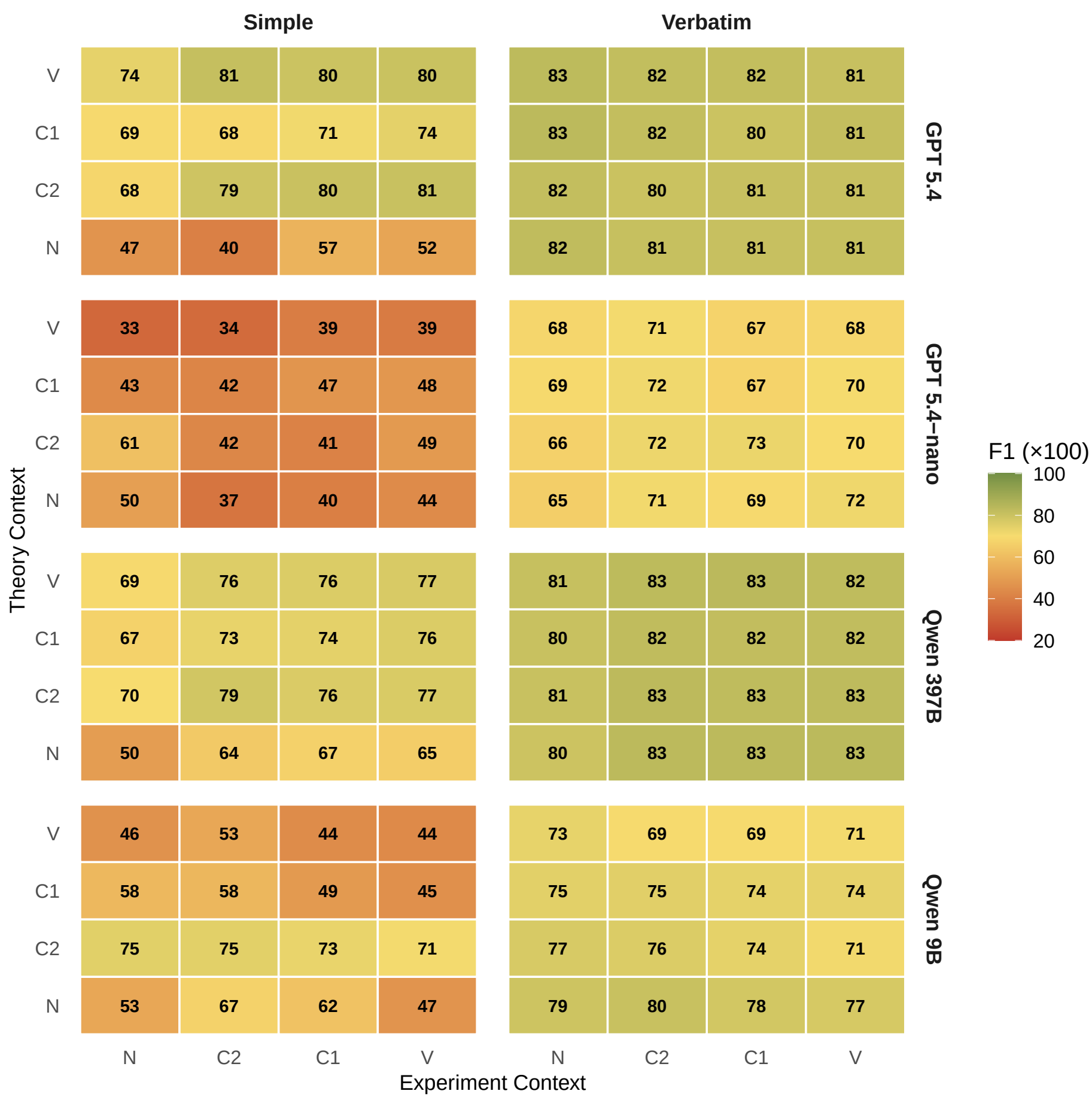

Note: F1 (×100) by experiment context (x-axis) and theory context (y-axis), separately under simple and verbatim classification instructions, for all four models. Compare to Figure 2.

Figure 1: L_{I} information surface (F1 metric).

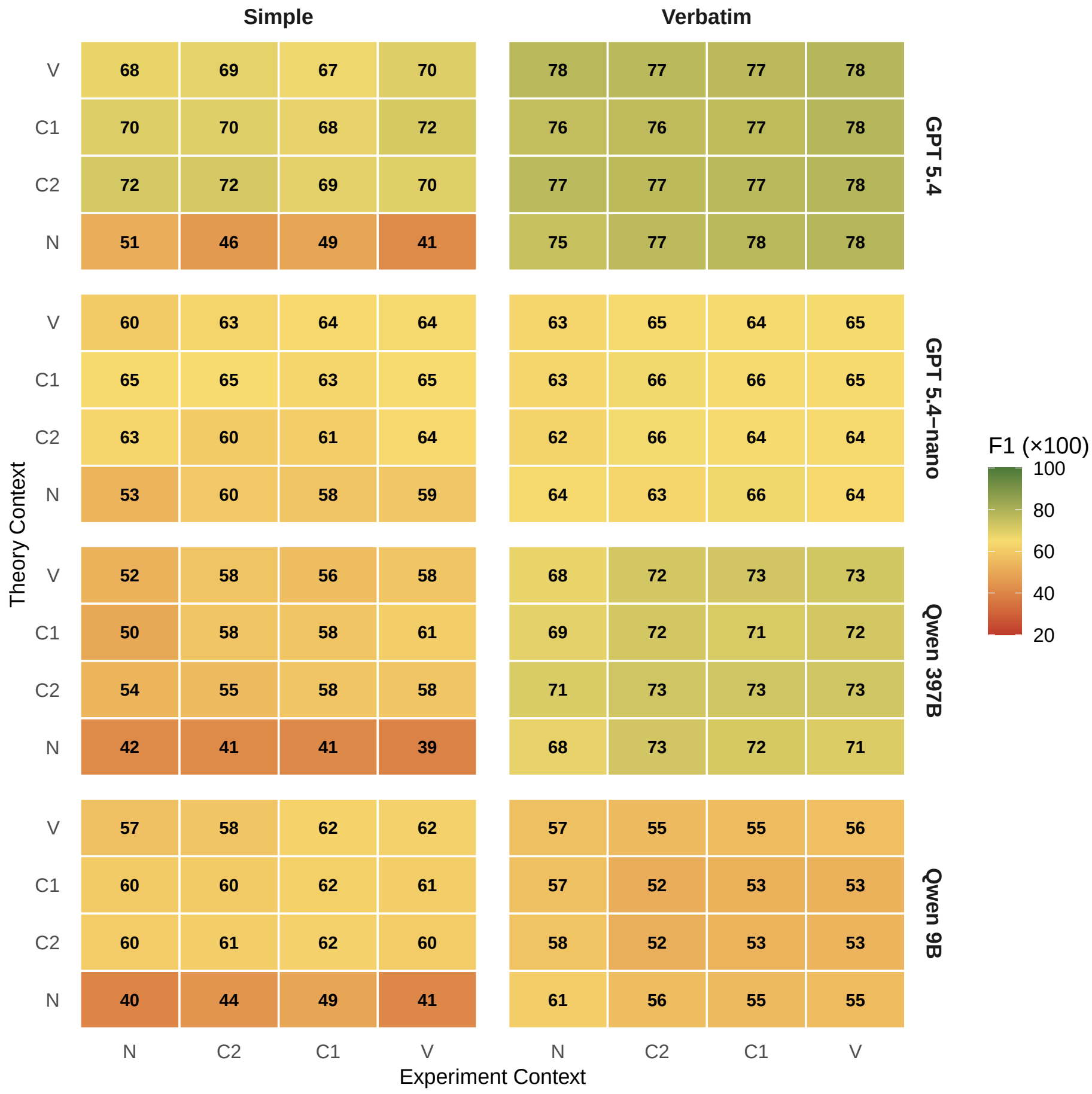

Note: F1 (×100) by experiment context and theory context for L_{II}, all four models. Compare to the L_{II} surface in Appendix 1.1.

Figure 2: L_{II} information surface (F1 metric).

1.7 Patterns and comparison with accuracy

Weighted F1 tracks the main-text accuracy numbers closely — same agreement protocol, same class weighting — so no bold ranking reverses and no main-text claim is overturned. Four patterns stand out across the eleven F1 tables.

1. Examples universally help. Δ_{Ex} is positive with a CI that excludes zero in all 12 model $\times$ task cells: $+.09$ to $+.19$ on P, $+.13$ to $+.38$ on L_{I}, $+.05$ to $+.26$ on L_{II}. The weakest effect is GPT 5.4-nano on L_{II} ($+.05$), still a real gain.

2. Content-vs-examples substitution scales with the task and the model. On P, $\Delta_{\mathrm{Content|Ex}}$ is essentially zero for every model: once examples are present, adding verbatim content adds nothing for the recognition-heavy task. On L_{II}, content still adds $+.07$ to $+.09$ for GPT 5.4, GPT 5.4-nano, and Qwen 397B even when examples are already present. Two model-specific exceptions stand out. Qwen 9B on L_{I} has $\Delta_{\mathrm{Content|Ex}} = +.17$ $[+.13, +.21]$ — the smallest model needs both full content and examples to reach its .82 ceiling. Qwen 9B on L_{II} has $\Delta_{\mathrm{Content|Ex}} = -.05$ $[-.08, -.01]$ —

the only cell where adding content on top of examples significantly hurts, suggesting the small model is saturated.

3. Reasoning cannot substitute for content, and its value drops with scale. $\Delta_{\text{Rea|Content}}$ is essentially zero for GPT 5.4 on every task, but becomes larger and reliably positive for smaller models on P ($+.05$ for nano, $+.10$ for Qwen 397B, $+.10$ for Qwen 9B). Qwen 9B is unique: reasoning-on-content is significant on all three tasks ($+.10$, $+.10$, $+.07$), meaning even with a full codebook and examples, the smallest model still extracts measurable benefit from reasoning.

4. Reasoning without content can actively hurt. Δ_{Rea} (reasoning added to the minimal prompt) is reliably negative on L_{I} for GPT 5.4 ($-.16$ $[-.21, -.11]$) and on L_{II} for every model except GPT 5.4 (nano $-.09$, Qwen 397B $-.02$ n.s., Qwen 9B $-.11$). Without classification instructions, reasoning leads the model toward plausible but wrong categorisations rather than correct ones.

Beyond these patterns, two observations about the metric itself are worth noting. On L_{I}, weighted F1 for frontier models tracks accuracy within a point (GPT 5.4: $.84$ vs. 84.0%; Qwen 397B: $.83$ vs. 83.6%), but for Qwen 9B it is several points below (.82 F1 vs. 82.8% accuracy), confirming that the smaller model concentrates its errors on minority levels. On L_{II}, weighted F1 is systematically a few points below interval accuracy for every model (GPT 5.4: $.78$ vs. 81.7%; Qwen 9B: $.61$ vs. 68.2%), reflecting uneven per-level recall: models often predict the same level within the interval and under-cover others. For aggregate agreement with human labels, interval accuracy is equivalent; for downstream analyses that depend on per-level reliability, weighted F1 is the more diagnostic metric.

References

[1]Camerer CF, Ho TH, Chong JK. A Cognitive Hierarchy Model of Games. The Quarterly Journal of Economics. 2004 August;119(3):861-98.

[2]Crawford VP, Costa-Gomes MA, Iriberri N. Structural Models of Nonequilibrium Strategic Thinking: Theory, Evidence, and Applications. Journal of Economic Literature. 2013 September;51(1):5-62. Available from: `http://www.aeaweb.org/articles.php?doi=10.1257/jel.51.1.5`. doi:10.1257/jel.51.1.5.
